# Structural and Functional Insights into a Honey Bee Omega-Class Glutathione S‑Transferase Mediating Chemical Sequestration and Antioxidative Stress

**DOI:** 10.1021/acs.jafc.6c03265

**Published:** 2026-05-28

**Authors:** Sonu Koirala, Timothy W. Moural, Gaurab Bhattarai, Ngoc T. Phan, Edwin G. Rajotte, David J. Biddinger, Fang Zhu

**Affiliations:** † Department of Entomology, 8082 Pennsylvania State University, University Park, Pennsylvania 16802, United States; ‡ Institute of Plant Breeding, Genetics & Genomics, University of Georgia, Athens, Georgia 30602, United States; § Department of Entomology and Plant Pathology, 3342 University of Arkansas, Arkansas 72701, United States; ∥ Research Center for Tropical Bees and Beekeeping, Gia Lam, Hanoi 100000, Vietnam; ⊥ Penn State Fruit Research and Extension Center, Biglerville, Pennsylvania 17307, United States; # Huck Institutes of the Life Sciences, Pennsylvania State University, University Park, Pennsylvania 16802, United States

**Keywords:** X-ray crystallography, cocrystal, enzymatic kinetics, pesticides, sequestration

## Abstract

The European honey bee (*Apis mellifera* L.) is an essential crop pollinator and is frequently exposed to pesticide residues that may compromise bee health. Mechanisms underlying chemical adaptation and detoxification in honey bees remain incompletely understood, particularly those involving glutathione S-transferases (GSTs). Here, we structurally and functionally characterized omega-class GST AmGSTO1. *AmGSTO1* was highly expressed in the fat bodies of nurse and forager bees. X-ray crystallography resolved the glutathione (GSH)-bound AmGSTO1 structure, revealing an active-site cysteine characteristic of omega GSTs. Enzyme assays showed greater catalytic efficiency toward the thioltransferase substrate HED than toward CDNB or PNA. Disc diffusion and bacterial survival assays demonstrated antioxidant activity against cumene hydroperoxide, hydrogen peroxide, and paraquat. Fluorescence binding assays indicated agrochemical binding, while HPLC detected no significant substrate depletion, suggesting a sequestration rather than catalytic role. Overall, AmGSTO1 may contribute to the protection against agrochemical toxicity and oxidative stress in honey bees.

## Graphical abstract

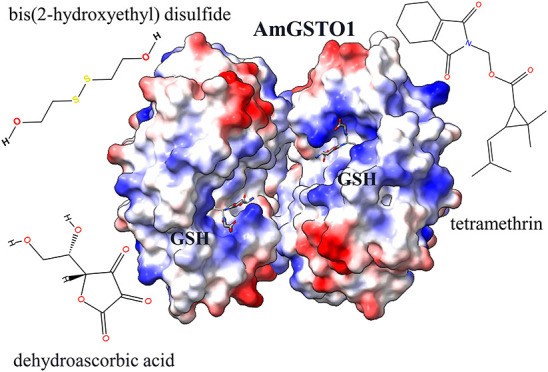

## Introduction

Honey bees have made significant ecological and economic contributions to human civilization through both their hive products and pollination services.^1,2^ However, in recent decades, several potential threats that directly impact honey bee health and their ecosystems have been identified, including pesticides, climate change, nutrient deficits, pests, pathogens, and their interactions.^3–8^ These threats ultimately lead to reduced agricultural productivity.^9^ Among different stressors, there is substantial evidence that honey bees are exposed to a variety of agrochemicals, both within their hive surroundings and through pollen and nectar that they consume.^10^ Research has revealed that, on average, North American bee colonies are contaminated with 6.5 different pesticides.^11^ Depending upon the route of exposure, mode of action, and doses, pesticides can be acutely toxic, chronically toxic, or nontoxic to bees.^12–14^ Additionally, bees that consume plant allelochemicals in their diet may enhance their ability to detoxify pesticides, thereby improving their resilience to chemical stress.^15,16^ Therefore, it is important to understand the molecular mechanisms by which bees adapt to potential toxins in the environment.^14^ Compared to other insects, honey bees have a lower number of detoxification enzymes, which may lead to greater sensitivity toward pesticides and other toxins in the environment.^17^ Nevertheless, they are still capable of metabolizing a variety of insecticides through enzymatic mechanisms, although the full range of chemicals they can process remains poorly characterized.^18^

Similar to other insects, the honey bee genome is comprised of numerous detoxification genes, including glutathione S-transferases (GSTs), a group of enzymes that play an important role in the detoxification of numerous endogenous and exogenous compounds.^17,19,20^ In general, GSTs catalyze the conjugation of a wide range of electrophilic substances and toxic compounds to glutathione (GSH). This process facilitates metabolism, detoxification, and elimination of numerous pesticides and plant toxins.^21,22^ Besides their roles in direct metabolism, GSTs may also participate in passive noncatalytic binding of substances (i.e., sequestration).^22,23^ Additionally, they exhibit peroxidase activity, helping to reduce oxidative stress caused by biotic and abiotic stressors.^20,24^ Insect cytosolic GSTs have been characterized and classified into six subclasses: delta, epsilon, omega, sigma, theta, and zeta.^25–28^ The honey bee genome contains 10 cytosolic GST genes. AmGSTO1 (NCBI accession number: XP_006569695.1) was identified as an omega-class GST in a previous study using sequence homology and phylogenetic approaches.^17^ Omega-class GSTs have been reported in varying numbers in other insect genomes such as one in mosquito (*Anopheles gambiae*), five in fruit fly (*Drosophila melanogaster*), two in pea aphid (*Acyrthosiphon pisum*), three in red flour beetle (*Tribolium castaneum*), and four in silk moth (*Bombyx mori*).^17,29,30^ Omega GSTs are cysteine-type GSTs, with a cysteine residue that interacts with GSH by forming a disulfide bond.^31^ In contrast, other cytosolic GSTs use tyrosine and serine for GSH stabilization and as the key residues in the active site necessary for metabolism of toxic chemical compounds.^26,32^ In addition, Omega GSTs have been linked to red eye development via the metabolism of eye pigments in *D. melanogaster* Meigen, demonstrating that omega-class GSTs function in biosynthetic pathways.^33^ In humans, the omega-class GSTs may have a broader role in ion channel modulation beyond their well-established functions in metabolism and detoxification.^34^ Some previous studies have suggested the potential involvement of insect omega-class GSTs in xenobiotic detoxification and insecticide resistance. For example, Yamamoto et al. reported that BmGSTO exhibits catalytic activity toward 4-hydroxynonenal, a product of lipid peroxidation in *B. mori*. In addition, BmGSTO showed high affinity for organophosphorus insecticides and was abundantly present in a silk moth strain exhibiting fenitrothion resistance.^35^ Balakrishnan et al. showed that *Escherichia coli* overexpressing *Rhopalosiphum padi* *RpGSTO1* exhibited increased resistance to cumene hydroperoxide–induced oxidative stress. The enzyme also displayed reduced GSH-dependent conjugating activity toward 1-chloro-2,4-dinitrobenzene (CDNB), and its expression was significantly altered following exposure to multiple insecticides, suggesting a potential role in detoxification.^36^ In a cyetpyrafen-resistant strain of *Panonychus citri*, *PcGSTO1* was reported to be significantly upregulated across developmental stages. Silencing *PcGSTO1* increased susceptibility to cyetpyrafen, and the recombinant enzyme was capable of metabolizing the acaricide.^37^ However, in pollinators, studies on omega-class GSTs remain limited, and little is known about their structural characteristics, substrate specificity, or roles in xenobiotic adaptation.

In this study, we investigated the structure and function of AmGSTO1 to elucidate its role in xenobiotic adaptation in honey bees. We characterized its tissue-specific expression, resolved its three-dimensional cocrystal structure with GSH by X-ray crystallography, and assessed its antioxidative properties. We further quantified its binding affinities to a broad panel of pesticides, metabolites, and plant allelochemicals and evaluated its catalytic activity toward selected agrochemicals using high-performance liquid chromatography (HPLC). Together, these analyses provide a comprehensive view of an omega-class GST in honey bees and highlight its potential role in pesticide sequestration and the oxidative stress response.

## Materials and Methods

### Phylogenetic Analysis of AmGSTO1 and Other Insect GSTs

The full coding sequence of the AmGSTO1 gene was cloned using sequence information from the NCBI database (accession number: XP_006569695.1) and primers listed in Table S1. PCR-amplified cDNA was purified, T4-treated, ligated into a *Hpa*I-digested pET-9Bc vector, and transformed into DH5α cells, and positive colonies were verified by plasmid extraction and sequencing (Functional Biosciences, WI, USA). To classify AmGSTO1, a phylogenetic tree was constructed using 157 GST amino acid sequences (Table S2) from five insect species (characterized and predicted) retrieved from NCBI: *A. mellifera*, *D. melanogaster*, *T. castaneum*, *Apis cerana cerana*, and *Bombus impatiens*. Multiple protein sequence alignment was performed using ClustalW^38^ in MEGA X^39^ with default parameters (gap open penalty: 10, gap extension penalty: 0.2). The maximum likelihood^40^ unrooted phylogenetic tree was inferred using RaxML 8.2.12 ^41^ under the PROTGAMMABLOSUM62 model with 500 bootstrap replicates. The model incorporates the BLOSUM62 substitution matrix and a gamma distribution to account for rate heterogeneity among sites.

### RNA Extraction, cDNA Synthesis, and qRT-PCR

Nurse and forager bees were collected from three hives spaced 60 m apart at the Penn State Wiley Apiary in University Park, PA, which were managed using standard best practices (https://extension.psu.edu/best-management-practices-for-bee-health; accessed on 20 December 2025). Nurse bees were taken from the brood nest, and foragers were collected by shaking honey frames.^42^ Then, the bees were flash frozen in liquid nitrogen and stored at −80 °C. Frozen nurse and forager bees were dissected in phosphate-buffered saline solution (0.01 M, pH 7.4), and the heads, fat bodies, Malpighian tubules, midguts, legs, and muscles were collected. Total RNA was extracted using Invitrogen TRIzol reagent (Invitrogen, Carlsbad, CA, USA) following the manufacturer’s protocol. For whole-body analysis, a biological replicate contained four nurse or forager bees, which were ground separately in liquid nitrogen prior to RNA extraction. For tissue analysis, each replicate comprised pooled tissues from 5–15 bees. Three biological replications were used for each experiment. RNA quality and purity were assessed with a NanoDrop One spectrophotometer (Thermo Fisher Scientific Inc., Waltham, MA, USA) using an A260/280 ratio of 1.8–2.0 as the quality criterion. cDNA synthesis was performed with M-MLV Reverse transcriptase (Thermo Fisher Scientific, Waltham, MA) and then cDNA was used as a template for qRT-PCR reactions. Each 10 μL reaction contained 1 μL of cDNA, 5 μL of FastStart SYBR Green Master (Roche Diagnostics, Indianapolis, IN, USA), 0.4 μL of qRT-PCR primers (Table S1), and 3.6 μL of ddH_2_O. Reactions were run on a Bio-Rad CFX Connect Real-Time PCR System (Bio-Rad, CA, USA). The thermal cycling conditions were 95 °C for 10 min, followed by 39 cycles of 95 °C for 10 s and 55 °C for 30 s. The most stable housekeeping gene, GAPDH (Glyceraldehyde-3-phosphate dehydrogenase) (XM393605), was used for the normalization of *AmGSTO1* expression using the 2-ΔΔCT method.^43,44^ The overall difference in the level of expression among the tissues was analyzed by one-way ANOVA with the Tukey HSD test for multiple comparisons in R (Version 4.1.0). The difference between the nurse and forager whole body expression was compared by Student’s *t* -test.

### Recombinant AmGSTO1 Expression and Purification

Honey bee AmGSTO1 (XP_006569695.1) with a 6×His tag was ligated into the circular plasmid vector (Addgene #48285 plasmid, pET-9Bc) via ligation-independent cloning. Then, the newly constructed plasmid was transferred into the Rosetta^TM^ II (DE3) pLysS BL21 *E. coli* expression strain. For protein expression, *E. co* *li* containing pET-9Bc-AmGSTO1 was incubated at 37 °C overnight at 250 rpm in 100 mL terrific broth (TB) media with antibiotics ampicillin (200 μg/mL) and chloramphenicol (CAM) (30 μg/mL).^45^ Then, the culture was inoculated in 2 L of TB media, which underwent incubation at 37 °C until its optical density (OD) at 600 nm reached between 0.4 and 0.6. AmGSTO1 expression was induced by the addition of 0.5 mM Isopropyl β-D-1-thiogalactopyranoside (IPTG) and incubated for another 24 h at 20 °C by shaking at 250 rpm. Cell pellets were harvested by centrifuging at 4000 rpm, 4 °C, and stored at −20 °C. AmGSTO1 was purified as described previously with some modifications.^44^ In brief, frozen cell pellets were resuspended in lysis buffer containing 25 mM NaPi, 500 mM NaCl, 3.3 mM NaN_3_, and 20 mM imidazole at pH 7.6, with 1 mM PMSF, 1 mM DTT, and a protease inhibitor tablet (Thermo Scientific), then lysed by sonication (Branson Digital Sonifier SFX 150). The lysate was centrifuged at 18,500 rcf and 4 °C to separate soluble protein and insoluble materials. The soluble protein was injected into an NGC Medium-Pressure Liquid Chromatography (MPLC) System (Bio-Rad Laboratories, Hercules, CA, USA) connected to a 5 mL Co-NTA column. Then, 6×His-tag fused AmGSTO1 was eluted with 20 mM NaPi, 300 mM NaCl, 3.3 mM NaN_3_, and 250 mM imidazole at pH 7.6. The resulting protein was subjected to a 100-fold buffer exchange, using a centrifugal concentrator with a molecular weight cutoff (MWCO) of 10 kDa, into a buffer solution containing 5 mM NaPi, 5 mM HEPES, and 5 mM DTT at pH 7.6. Buffer-exchanged protein was further purified by a Hydroxyapatite column (HA, ceramic hydroxyapatite type I, Bio-Rad) connected to an NGC MPLC system. Then, the gradient ranging from 5 mM NaPi, 5 mM HEPES, and 5 mM DTT at pH 7.6 to 500 mM NaPi and 3.3 mM NaN_3_ at pH 7.6 was used to wash and elute the recombinant protein. The protein was buffer exchanged with a solution containing 20 mM Tris at pH 7.6 using a centrifugal concentrator (10 kDa MWCO) and then introduced to an Enrich Q 10x100 mm high-resolution ion exchange column (BIO-RAD). The gradient ranging from 20 mM Tris at pH 7.6 with 2 mM DTT to 20 mM Tris, 1 M NaCl, and 2 mM DTT at pH 7.6 was used to wash and elute the protein. Then, the final purification step was performed with a Cytiva HiPrep Sephacryl S-200 HR size exclusion column with 20 mM HEPES, 150 mM NaCl, 1 mM EDTA, and 20 mM GSH at pH 7.6. Protein obtained from various columns was analyzed by SDS-PAGE and concentration determination using a NanoDrop^TM^ One (Thermo Fisher Scientific) spectrophotometer to validate the quality and quantity of protein (Figure S1). The protein was flash-frozen in liquid nitrogen and stored at −80 °C for future use.

### X-ray Crystallography of AmGSTO1-GSH Complex and Molecular Docking with CDNB

AmGSTO1 was crystallized by hanging drop vapor diffusion at 18 °C. Protein (5 mg/mL) with 20 mM GSH was mixed 1:1 with a reservoir solution (0.2 M ammonium acetate and 20% PEG 3350) in VDX plates. The cocrystals were placed into cryoprotection solution (0.2 M ammonium acetate, 20% PEG 3350, 30% glycerol), and X-ray diffraction data were collected at the Advanced Photon Source Structural Biology Centers beamline 19-ID. Software such as Collaborative Computational Project Number 4 (CCP4) with DIALS and xia2 was used for processing diffraction data.^46–50^ Phaser implemented in Phenix was used for phasing using an AlphaFold2 model as a search model for molecular replacement.^51–53^ Model building and refinement were performed using Phenix and Coot.^54,55^ Structural analysis and figures were generated following previously published methods.^44^ For molecular docking, the structure of CDNB was downloaded from the PubChem database in SDF format.^56^ Ligands were docked using AutoDock Vina in UCSF Chimera, with the prepared chain A structure (GSH-bound) used as the receptor.^57,58^ The substrate-binding pocket was used as the docking site. Nine poses were generated for CDNB, and one representative pose was selected based on the AutoDock Vina docking score, with more negative scores indicating stronger binding affinity. Figures with docked ligands were prepared in ChimeraX.^59^

### Enzymatic Assay

To understand the enzymatic property and its substrate range, the kinetics analysis of purified recombinant AmGSTO1 was conducted using various substrates, including 2-hydroxyethyl disulfide (HED), CDNB, reduced GSH, and p-nitrophenyl acetate (PNA), and dehydroascorbic acid (DHA).^60–62^ Stock solutions (100 mM) of CDNB and PNA were prepared in ethanol, DHA in DMSO, and GSH in a KPi buffer. CDNB-conjugating activity was assayed by varying CDNB concentrations (0.125–1.25 mM) at constant 1 mM GSH. PNA activity was measured with substrates ranging from 0.0625 to 1.5 mM (GSH fixed at 0.5 mM). GSH assayed using 0.031–0.75 mM GSH and CDNB fixed at 1 mM. DHA reductase activity was measured using DHA at 1 mM and 1 mM GSH. The concentration of AmGSTO1 was 0.2 mg/mL for CDNB and GSH and 0.8 mg/mL for PNA and DHA, in 100 mM KPi buffer (pH 8). Thioltransferase activity was measured based on methods from Whitbread et al. 2005 and Poirier et al. 2024.^63,64^ Briefly, the coupled reaction contained 1 μM AmGSTO1, 0.5 units of glutathione reductase (yeast), 2 mM GSH, 100 μM NADPH, and variable HED concentrations from 8 to 256 μM with the reaction buffer 100 mM Tris, pH 8.0. Reactions were carried out in 96-well UVstar microplates (Greiner Bio-One). The assays were conducted in triplicate with a reaction volume of 200 μL. Absorbance at 340 nm (CDNB), 405 nm (PNA), 265 nm (DHA), and 340 nm (NADPH) was monitored for 1 min 10 s using a Spark multimode plate reader (TECAN, Mannedorf, Switzerland). Enzyme-free controls were included to correct for non-enzymatic reaction. Kinetic parameters were calculated using published molar attenuation coefficients, path length corrected for 96-well plates, and analyzed in GraphPad Prism.^21,60,61,63,64^

### Site-Directed Mutagenesis

To examine the contribution of active-site residues to enzyme function, Cys28, Tyr30, Glu81, Ser82, Phe127, and Trp174 of AmGSTO1 were mutated to Ala. PCR primers were designed using NEBaseChanger (New England Biolabs; https://nebasechangerv1.neb.com/) to introduce specific nucleotide substitutions or modifications for AmGSTO1 (Table S1). Amino acid substitution variants of AmGSTO1 were generated utilizing the Q5 Site-Directed Mutagenesis Kit (New England Biolabs, MA, USA). Wild-type recombinant AmGSTO1-pET-9Bc was used as the template for generating mutants, and full-length mutant constructs were verified with DNA sequencing. Mutant AmGSTO1-pET-9Bc was introduced into Rosetta II (DE3) pLysS cells for expression, and proteins were purified using the same procedure as wild-type AmGSTO1. Purified mutant AmGSTO1 protein was then subjected to GSH-CDNB kinetic assay, as described in the previous section using mutant AmGSTO1 at a concentration of 0.8 mg/mL.

### Fluorescence Binding Assay

Fluorescence binding assay was performed to examine H-site interactions of GSTs using 8-Anilinonaphthalene-1-sulfonic acid (ANS) as the probe (excitation: 380/20 nm, emission: 485/20 nm bandwidth). ANS saturation binding assay was performed with AmGSTO1 to determine the maximum binding capacity (*B*_max_) and equilibrium dissociation constant (*K*_d_). Two micromolar AmGSTO1 was titrated with 0–225 μM ANS, and the curve was fitted in GraphPad Prism 9.5.1. The ANS displacement assay assessed competition by other substrates. For the displacement assay, AmGSTO1 (2 μM) and ANS (50 μM) were incubated with competitor ligands (0–4 mM) in 20 mM potassium phosphate buffer containing 150 mM NaCl, 1 mM EDTA, and 1 mM Tris (2-carboxyethyl) phosphine (TCEP) (pH 6) in 200 μL reactions in 96-well black plates. Measurements were performed using a Tecan Spark multimode plate reader, with CDNB and ethacrynic acid (EA) as positive controls and GSH as a negative control for ANS displacement. In agricultural and urban environments, bees are exposed to complex mixtures of agrochemicals, including herbicides, fungicides, insecticides, and their metabolites, which have been detected in beeswax, pollen, and bee samples.^11,15,65–69^ Similarly, bees are also naturally exposed to plant secondary metabolites present in nectar and pollen, including alkaloids, phenolics, and isothiocyanates.^16,70,71^ Therefore, representative compounds from multiple pesticide classes and plant allelochemicals were selected for the ANS binding assay to evaluate the binding specificity of AmGSTO1 toward ecologically relevant xenobiotics. Multiple herbicides (atrazine, clopyralid, 2,4-D, dicamba, fenoprop, metolachlor, paraquat, picloram, 3,5,6-trichloro-2-pyridinol [TCP], triclopyr), fungicides (chlorothalonil, propiconazole, pyraclostrobin, myclobutanil), insecticides and their metabolites (acephate, amitraz, bifenthrin, carbaryl, 6-chloronicotinic acid, chloropyrifos, coumaphos, deltamethrin, diazinon, dinotefuran, fenvalerate, imidacloprid, malathion, metamidophos, permethrin, 3-phenoxybenzaldehyde, tetramethrin), and plant allelochemicals (caffeine, cotinine, nicotine, p-coumaric acid, phenethyl isothiocyanate, phenylacetaldehyde, propyl isothiocyanate) were used as competitors in ANS displacement assays. Each reaction was performed in triplicate. Finally, IC_50_ values for competitive ligands were calculated using GraphPad Prism 9.5.1, and dissociation constants (*K*_i_) were determined using the equation *K*_i_ = [IC_50_]/(1 + [ANS]/*K*_d_^ANS^).^72^

### Metabolism Assay by HPLC MS/MS

To assess the catalytic ability of AmGSTO1, the enzymatic assay for the HPLC study was conducted in a reaction containing 0.02 M ammonium acetate, 4 mM GSH, 0.2 mM of each pesticide (pesticides were dissolved in methanol to make 100 mM stock solutions), and 2 μM of the enzyme in a total volume of 500 μL. Following a 1 h incubation, the reaction was halted by the addition of 500 μL of methanol. Subsequently, the mixture underwent separation using 3K centrifugal filter units. The filtrate was then introduced into the high-sensitivity Vanquish Flex UHPLC system for LC-MS and LC-MS/MS analyses located in the Huck Core Facility (Penn State University). A non-enzymatic reaction control was carried out, comprising the same amount of heat-inactivated enzyme and all other reactants used in treatment groups. Each reaction, for both controls and treatments, was performed in triplicate (*n* = 3). The chemicals included fenoprop, TCP, and 2,4-D. Detection of chemical constituents was achieved through a UV detector, while the obtained mass spectra were cross-referenced with the NIST Mass Spectral Database version 2.0 (NIST, Gaithersburg, MD) to elucidate the chemical composition within the mixture. Differences in mean peak area between control and treatment groups were analyzed using a two-tailed Student’s *t*-test.

### Disc Diffusion Assay

To access the antioxidative properties of AmGSTO1, a disc diffusion assay was performed with a method modified from Burmeister’s protocol.^73^ Transformed cells with recombinant AmGSTO1-pET-9Bc plasmid were used as the treatment, and the empty plasmid (pET-9Bc) vector served as a control. For control and treatment, transformed Rosett II (DE3) pLysS cells were grown in lysogeny broth (LB) with ampicillin (200 μg/mL) and chloramphenicol (30 μg/mL) at 37 °C, 250 rpm. When culture reached OD_600 nm_ = 0.6, cells were induced with 1 mM IPTG. Next, the cultures were incubated at 37 °C for 7 h. Approximately 5 × 10^8^ cells were plated on LB agar with ampicillin (200 μg/mL) and chloramphenicol (30 μg/mL). Then, the plates were incubated at 37 °C for 1h to dry. After that, filter discs (6 mm diameter) soaked with different concentrations (0, 50, 100, 150, and 200 mM) of cumene hydroperoxide (CHP) and hydrogen peroxide (H_2_O_2_) were placed on the plates separately. Then, after 12 h of incubation at 37 °C, the halo diameter of control and treatment plates was recorded. All assays were performed in triplicate. The differences in the diameter of the halo were analyzed by one-way ANOVA with the Tukey HSD test for multiple comparisons in R (Version 4.1.0).

### Bacterial Survival Assay

To assess the role of AmGSTO1 against oxidative stress, a bacterial survival assay was performed following Dong’s protocol.^74^ H_2_O_2_ and paraquat were dissolved in water. *E. coli* carrying an empty plasmid (pET-9Bc) vector was used as the control, and cells with recombinant AmGSTO1-pET-9Bc were used as the treatment. Treatment and control cells (5 mL) were cultured separately in 2YT media containing ampicillin (200 μg/mL) and CAM (30 μg/mL) at 37 °C, 180 rpm. When cultures reached OD_600 nm_ = 0.3, 1 mM IPTG and 0.5 mM H_2_O_2_ or paraquat were added to the control or treatment cells. After 1 h of induction with oxidative agents at 37 °C, 180 rpm, the OD_600 nm_ was measured hourly. After 7–8 h of incubation, bacterial suspensions were diluted 10,000-fold in autoclaved water, and 50 μL of the bacteria was spread on LB agar plates containing ampicillin (200 μg/mL) and CAM (30 μg/mL). Colony-forming units (CFUs) were recorded after overnight incubation at 37 °C. All assays were repeated with three biological replicates, and differences between control and treatment groups were analyzed using Student’s *t*-test.

## Results

### Phylogenetic Relationships of AmGSTO1 with Other Insect GSTs

The phylogenetic analysis of AmGSTO1 (accession number: XP_006569695.1) was performed to investigate its evolutionary relationships (Table S2) and establish orthology with other insect GSTs. The AmGSTO1 sequence has an open reading frame of 726 bp, encoding a 242-amino-acid protein. Phylogenetic analysis grouped GST enzymes by class. AmGSTO1 clustered within the omega clade alongside an omega-class GST from *A. cerana cerana* (AccGSTO1; XP_016918436.1) and its predicted isoform (AGX26610.1), as well as an omega-class GST from *B. impatiens* (XP_012237875.1) (Figure 1).

**Figure 1:**
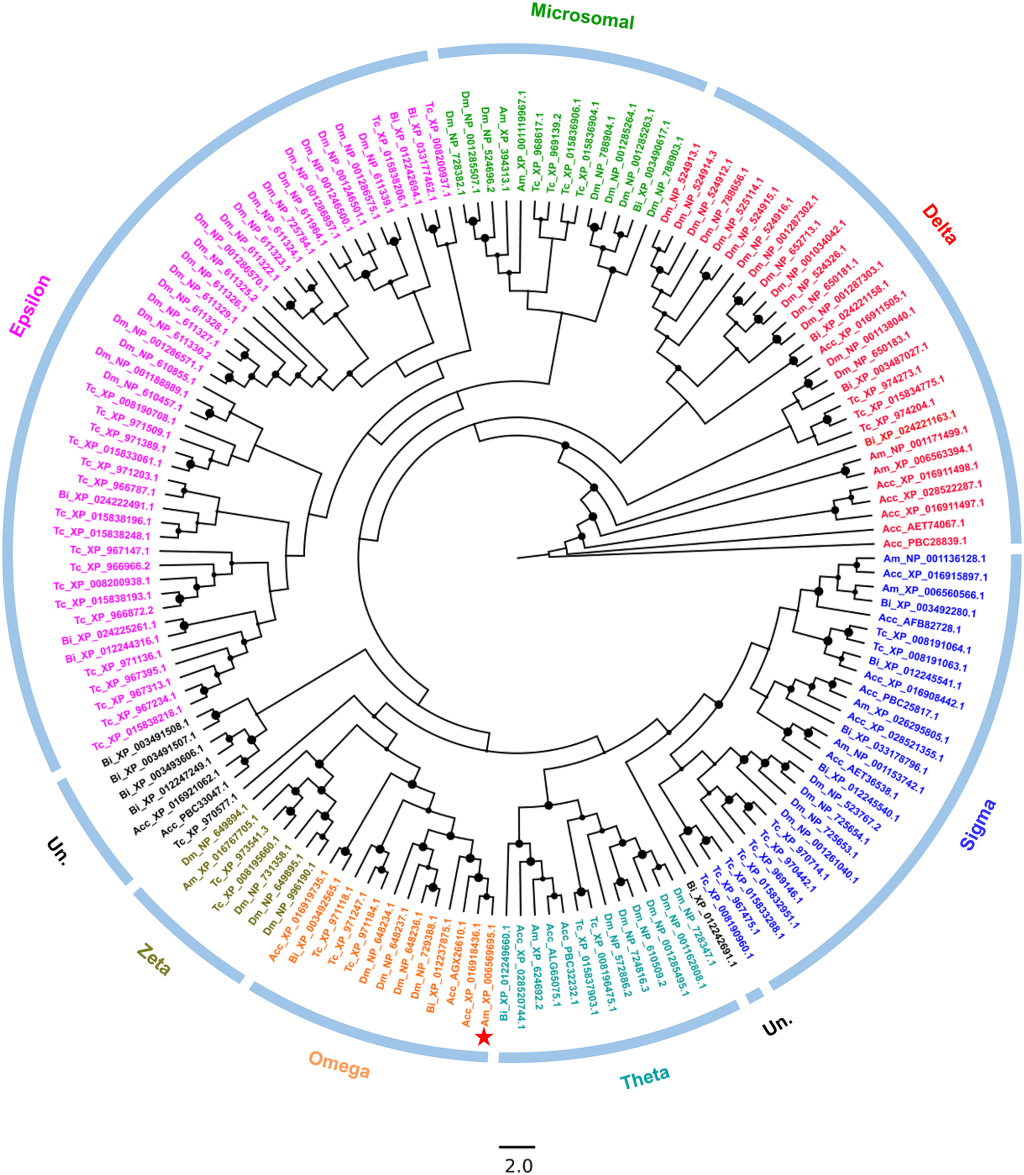
Unrooted maximum likelihood tree of 157 GST proteins in five different insect species: *D. melanogaster* (Dm), *T. castaneum* (Tc), *A. mellifera* (Am), *A. cerana cerana* (Acc), and *B. impatiens* (Bi). The color of the terminal nodes represents cytosolic GST from various classes. GST proteins in black indicate uncharacterized ones. The size of solid circles at each node represents the bootstrap support value. Accession indicated with a star symbol indicates AmGSTO1.

### Gene Expression Patterns of *AmGSTO1* in Nurse and Forager Bees

qRT-PCR analysis was carried out to examine the temporal and spatial expression patterns of *AmGSTO1* in the nurse and forager bees. The relative expression of *AmGSTO1* in forager bees was significantly higher than that in nurse bees (*p* < 0.001) (Figure 2A). In terms of spatial expression, we found a significant difference in *AmGSTO1* expression among different tissues in both nurse (*p* < 0.001; df = 5; *F* = 113.52) and forager bees (*p* < 0.001; df = 7; *F* = 30.96). Specifically, in nurse bees, the mean relative expression of *AmGSTO1* was significantly higher in the fat body, followed by the Malpighian tubule, head, midgut, leg, and muscle (Figure 2B). In forager bees, the expression of *AmGSTO1* was significantly higher in the fat body, followed by the hind leg, Malpighian tubule, fore leg, and head, with comparatively lower expression in the middle leg, midgut, and muscle (Figure 2C).

**Figure 2:**
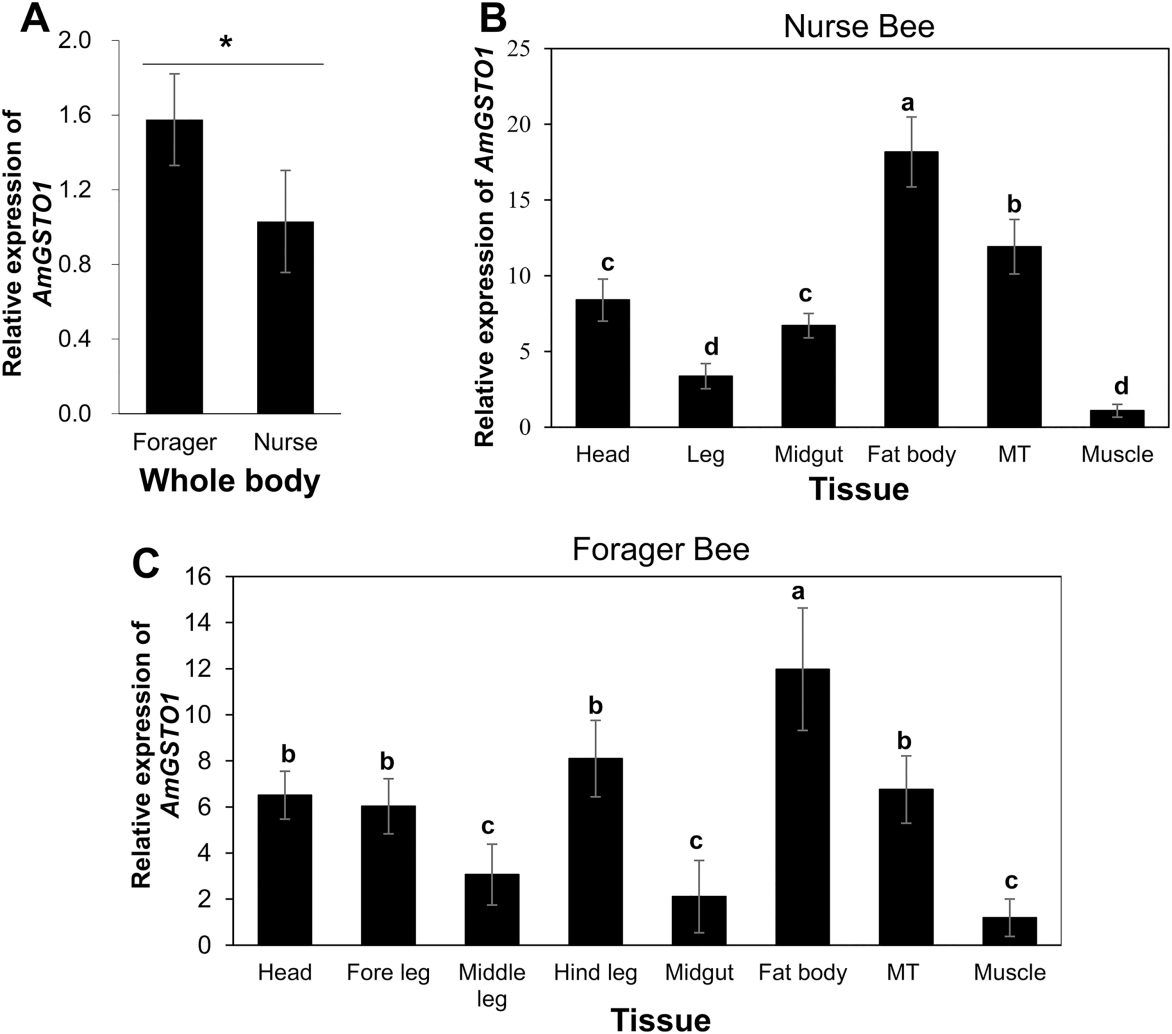
Gene expression patterns of *AmGSTO1* in nurse and forager bees. (A) Whole body gene expression pattern. Spatial expression profile in nurse bees (B) and forager bees (C). Different letters indicate a significant difference in gene expression at *p* < 0.001, according to one-way ANOVA with the Tukey HSD test. * Indicates a significant difference in gene expression between nurse and forager bees by Student’s *t*-test analysis. Note: MT-Malpighian tubules.

### X-ray Crystal Structure of AmGSTO1 and Its Co-Crystal with GSH

A protein bank database (PDB) search with the AmGTSO1 sequence revealed that the highest sequence match with insects was *B. mori* omega-class GST (3WD6) with a sequence identity of 35%.^75^ The asymmetric unit contained four molecules of AmGSTO1, each complexed with one GSH molecule. AmGSTO1 crystallized in space group P 2_1_, where the unit cell dimensions were *a* = 64.14 (Å), *b* = 78.39 (Å), *c* = 107.97 (Å), with angles α = 90.00°, β = 106.70°, and γ = 90.00°. The structure was refined out to 2.05 (Å), with *R*_work_ and *R*_free_ values of 22.10 and 25.78%, respectively (Table 1). An analysis of the diffraction data indicated that AmGSTO1 forms a dimeric protein structure. The dimeric protein comprises two monomers designated as subunit 1 and subunit 2 (Figure 3). The AmGSTO1 monomer comprises N-terminal and C-terminal domains connected by a 10-amino acid linker. The structure exhibits a typical thioredoxin-like fold, with an N-terminal comprising β_1_α_1_β_2_α_2_β_3_β_4_α_3_ motifs and a C-terminal helical domain. The N-terminal domain was ordered starting with β_1_ (residues I20–S24), followed by α_1_ (residues P29-A40), β_2_ (residues H45–Y49), α_2_ (residues D57-K62), β_3_ (residues C70-E72), β_4_ (residues I78–Y80), and α_3_ (residues S82-T92). The C-terminal domain commenced with α_4_ (residues P103–I128), followed by α_5_ (residues Q134-R154), α_6_ (residues M166-R185) with a bulge at R177, α_7_ (residues D187-F189), α_8_ (residues K197-E208) with a bulge at N209, α_9_ (residues P210–N215), and α_10_ (residues T219-R230) (Figure 3). Each subunit contains two ligand-binding sites: a conserved G-site in Domain I for binding GSH and an H-site in Domain II for hydrophobic substrates.

**Figure 3:**
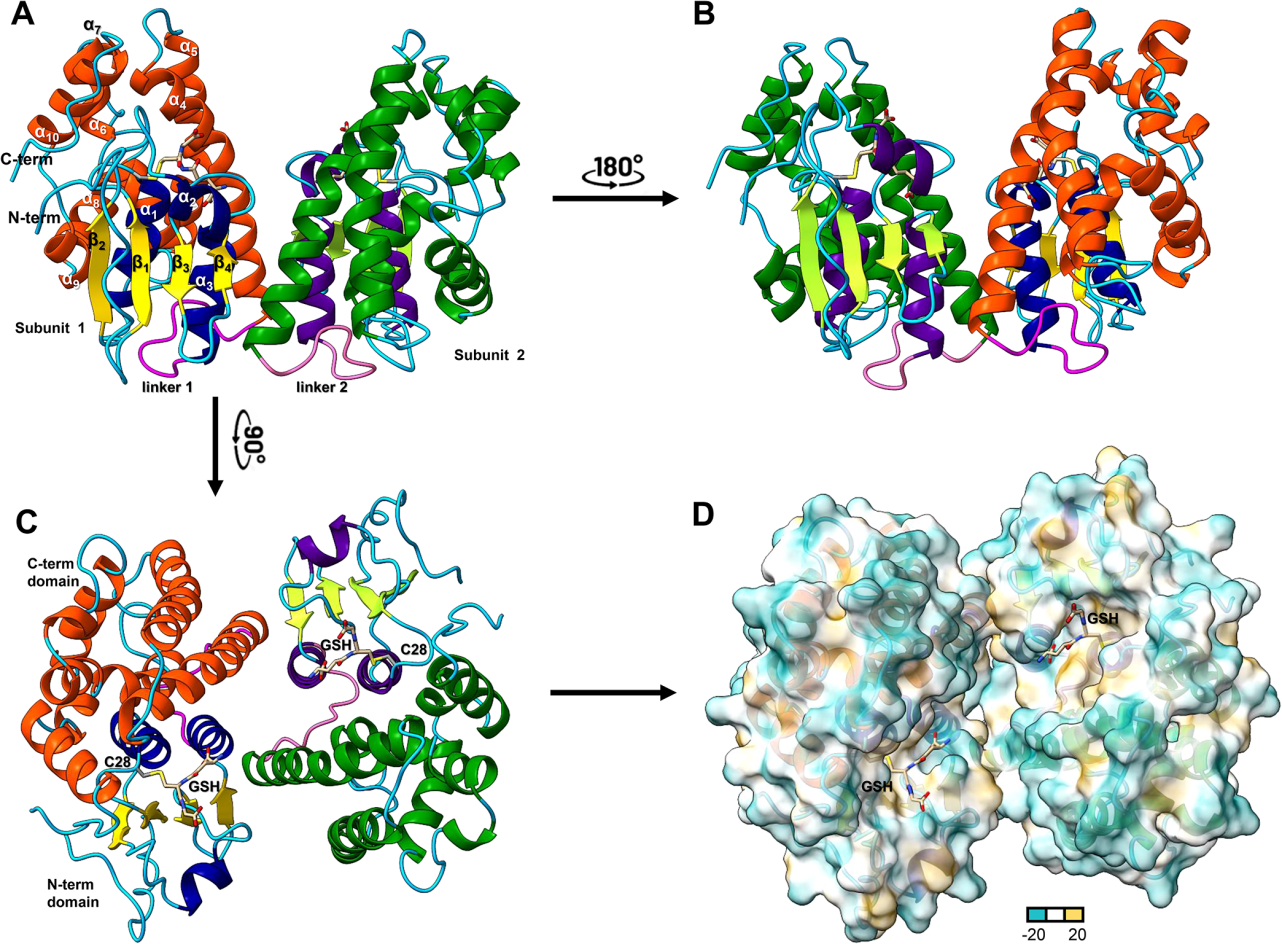
AmGSTO1-GSH cocrystal structure shown as a dimer and represented in a ribbon diagram and surface overlaid on a ribbon diagram. (A) Dimeric ribbon diagram of AmGSTO1 showing Subunit 1 (monomer 1) and Subunit 2 (monomer 2). (B) The ribbon diagram of the dimeric AmGSTO1 structure from A has been rotated 180°, showing the 2-fold symmetry of the dimer. (C) The ribbon diagram of the dimeric AmGSTO1 structure from A is rotated 90°. (D) The dimer ribbon diagram overlaid with the lipophilic surface. The lipophilic representation scale is shown with −20 being the least lipophilic and 20 being the most lipophilic. Figures were generated with UCSF Chimera X v-1.5.

**Table 1:** Data Collection and Refinement for AmGSTO1 with GSH^a^

AmGSTO1 complex with GSH		PDB: 10HA
Data Collection		
Wavelength (A)		0.9792
Resolution range (A)		61.09–2.05 (2.1–2.05)
Space group		P 1 21 1
Cell Dimensions		
a, b, c (Å)		64.12, 78.45, 107.97
α, β, γ (°)		90.00, 106.68, 90.00
Total reflections		201813 (14739)
Unique reflections		63482 (4529)
Multiplicity		3.2 (3.3)
Completeness (%)		98.40 (98.80)
Mean I/sigma(I)		6.09 (1.79)
Wilson B-factor		37.48
R-merge		0.089 (0.54)
R-meas		0.11 (0.64)
R-pim		0.059 (0.35)
CC1/2		0.97 (0.41)
CC*		0.99 (0.76)
Refinement		
Reflections used in refinement		63416 (4515)
Reflections used for R-free		1997 (139)
R-work		0.221 (0.300)
R-free		0.258 (0.334)
Number of non-hydrogen atoms		8021
Macromolecules		7768
Ligands		80
Solvent		136
Protein residues		948
RMS(bonds)		0.008
RMS(angles)		0.86
Ramachandran favored (%)		97.77
Ramachandran allowed (%)		2.23
Ramachandran outliers (%)		0.00
Rotamer outliers (%)		0.35
Clash score		3.90
Average B-factor		55.52
Macromolecules		55.82
Ligands		39.88
Solvent		49.16

a Numbers in parentheses are for the highest resolution shell.

### Active-Site Determination

The AmGSTO1-GSH cocrystal structure revealed a hydrophilic G-site in the N-terminal domain interacting with GSH, adjacent to a lipophilic pocket for hydrophobic substrate binding (Figure 3). Structural analysis indicates that the glutathionyl moiety of the ligand forms hydrogen bonds with residues Lys67, Val68, Glu81, and Ser82 with interaction distances ranging from 2.6 to 3.4 Å (Figure 4A). The sulfur atom from glutathione’s cysteine residue formed a mixed disulfide with the active-site cysteine-28 (Cys28) of AmGSTO1 (Figure 4A,C). Residues Glu81 and Ser82 participate in binding with the γ-glutamyl moiety of GSH. The Glu81 OE1 atom lies 3.01 Å from the GSH γ-glutamyl N1 atom, while the Ser82 OG and N atoms are positioned 2.92 Å from the γ-glutamyl O12 atom, respectively. Additional hydrogen bond interactions were between the glycyl O32 of GSH and Lys55 NZ of AmGSTO1 (2.74 Å), along with a hydrogen bond between the GSH cysteine carbonyl oxygen O2 and Val68 of AmGSTO1 (3.02 Å). The surrounding GSH-binding pocket is formed by Tyr30, Leu52, Lys55, Gly66, and Pro69. Among the generated docking poses, the CDNB with the lowest binding energy (−6.09 kcal/mol) was selected, with C3 located 4.68 Å from the glutathione sulfur atom. The H-site is located adjacent to the G-site, composed of Pro29, Tyr30, Cys124, Phe127, Ile128, Trp174, Arg177, Try225, Met226, and Arg229 (Figure 4B). Conserved omega-class GST residues in the G-site were found to be Cys28, Pro29, Arg33, and Leu38 (Figures 4C and S2).

**Figure 4:**
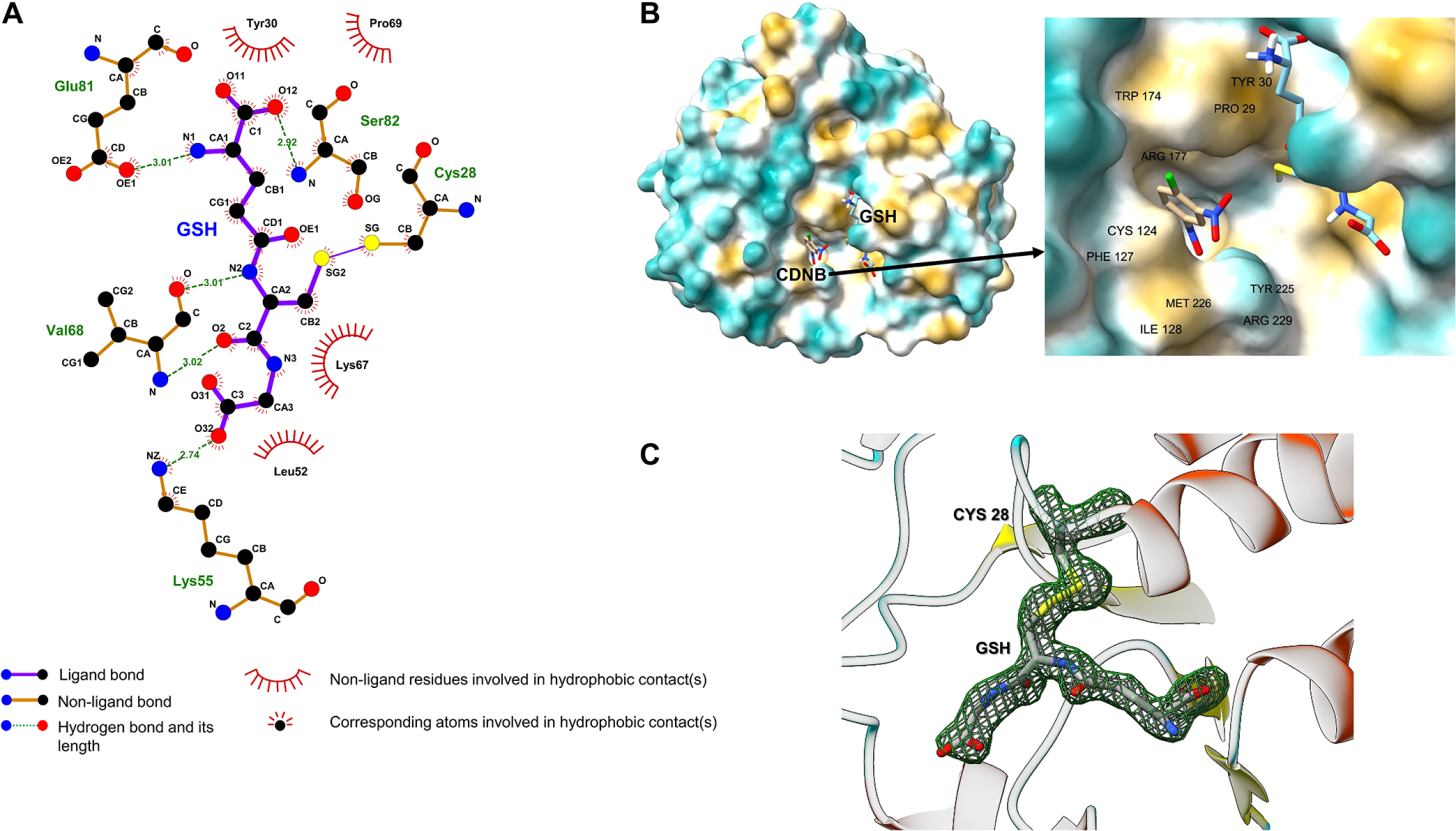
Protein–ligand interactions of AmGSTO1. (A) AmGSTO1-GSH interactions are represented graphically using LigPlot+.^110^ AmGSTO1 active-site amino acid residues at the G-site that bind GSH are shown. (B) AmGSTO1 active-site amino acid residues at the H-site that are in close contact with the docked ligand CDNB. (C) Ribbon diagram of AmGSTO1 zoomed in on the G-site to display electron density for CYS28-GSH mixed disulfide. Polder map (mFo-DFc_polder) is displayed to show density within 3.5 Å of GSH and contoured ± 3 σ.

### Enzymatic Kinetics of Wild-Type and Mutant AmGSTO1

The kinetic parameters *V*_max_, *K*_m_, *k*_cat_, and *k*_cat_/*K*_m_ of AmGSTO1 toward HED, CDNB, GSH, and PNA were determined. Parameters were calculated in GraphPad Prism 9.5.1 by fitting experimental data to the Michaelis–Menten model using nonlinear regression (Figure 5). For various concentrations of HED, CDNB, and PNA while keeping the GSH concentration constant, *V*_max_ values were 14.62 ± 0.81 μM/min, 30.21 ± 1.08 μM/min, and 57.45 ± 5.09 μM/min; *K*_m_ values were 40.88 ± 6.73 μM, 0.58 ± 0.05 mM, and 0.69 ± 0.14 mM; *k*_cat_ values were 14.62 ± 0.81, 4.50 ± 0.16, and 2.14 ± 0.19 min^–1^; and *k*_cat_/*K*_m_ values were 0.36 ± 0.06, 7.76 ± 0.72, and 3.10 ± 0.66 mM^–1^·min^–1^, respectively (Table 2). When varying the concentration of GSH while maintaining a constant concentration of CDNB, the *V*_max_, *K*_m_, *k*_cat_, and *k*_cat_/*K*_m_ were 13.27 ± 0.49 μM/min, 0.03 ± 0.01 mM, 1.98 ± 0.07 min^–1^, and 66.00 ± 22.14 mM^–1^·min^–1^, respectively (Table 2). The DHA reductase activity was determined at 1 mM DHA and 1 mM GSH. The initial velocity was 205.6 ± 1.9 μM/min, and the specific activity was 0.257 ± 0.002 μmol/min/mg. AmGSTO1 showed no detectable enzymatic activity toward isothiocyanate, 4-hydroxynonenal, and trans-2-hexenal (T2H).

**Figure 5:**
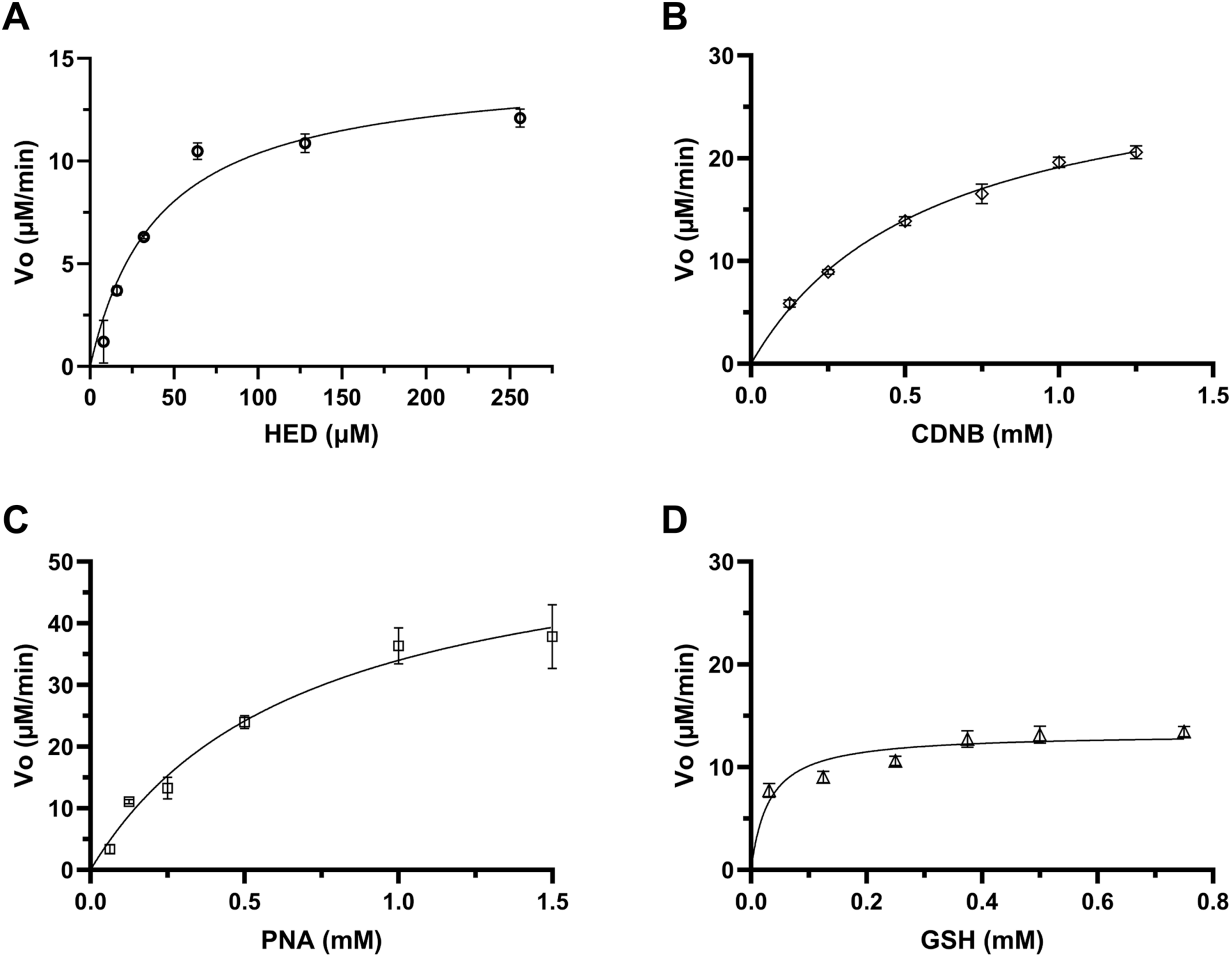
Plots of initial reaction velocity (*V*_o_) as a function of substrate concentration. (A) Thioltransferase activity on HED at constant GSH concentration and variable concentrations of HED from 8 μM to 256 μM. (B) The CDNB-conjugating activity at constant concentrations of GSH and various concentrations of CDNB from 0.125 mM to 1.25 mM. (C) The thiolytic activity toward PNA was tested using different concentrations of PNA ranging from 0.0625 mM to 1.5 mM, with the concentration of GSH held constant. (D) The GSH-conjugation activity at various concentrations of GSH, from 0.031 mM to 0.75 mM while holding the CDNB concentration constant. *K*_m_ and *V*_max_ were calculated for each substrate using GraphPad Prism 9.5.1 by fitting experimental data to a nonlinear regression curve to obtain the Michaelis–Menten plot.

**Table 2:** Kinetic Parameters for Wild-Type and Mutant AmGSTO1 (WT – Wild-Type)^a^

H-site (various concentrations of HED, PNA, and CDNB with constant GSH concentration)				
	*V*_max_ (μM/min)	*K*_m_ (μM)	*k*_cat_ (min^–1^)	*k*_cat_/*K*_m_ (μM^–1^ · min^–1^)
HED	14.62 ± 0.81	40.88 ± 6.73	14.62 ± 0.81	0.36 ± 0.06
	*V*_max_ (μM/min)	*K*_m_ (mM)	*k*_cat_ (min^–1^)	*k*_cat_/*K*_m_ (mM^–1^ · min^–1^)
PNA	57.45 ± 5.09	0.69 ± 0.14	2.14 ± 0.19	3.10 ± 0.66
CDNB				
WT AmGSTO1	30.21 ± 1.08	0.58 ± 0.05	4.50 ± 0.16	7.76 ± 0.72
Y30A	16.62 ± 0.72	0.21 ± 0.03	0.62 ± 0.04	2.95 ± 0.49
E81A	31.81 ± 1.60	0.86 ± 0.10	1.19 ± 0.06	1.38 ± 0.17
F127A	22.26 ± 1.89	0.64 ± 0.15	0.83 ± 0.07	1.30 ± 0.32
S82A	29.02 ± 2.06	0.76 ± 0.12	1.08 ± 0.08	1.42 ± 0.24
W174A	79.22 ± 13.22	2.28 ± 0.68	2.95 ± 0.49	1.29 ± 0.43

G-site (various concentrations of GSH and constant CDNB)				
	*V*_max_ (μM/min)	*K*_m_ (mM)	*k*_cat_ (min^–1^)	*k*_cat_/*K*_m_ (mM^–1^ · min^–1^)
WT AmGSTO1	13.27 ± 0.49	0.03 ± 0.01	1.98 ± 0.07	66.00 ± 22.14
C28A	263.85 ± 21.98	0.29 ± 0.06	9.84 ± 0.82	33.93 ± 7.58

a Values are reported as mean ± SE from nonlinear regression fitting with GraphPad Prism.

To assess the functional roles of active-site residues (Cys28, Tyr30, Ser82, Phe127, Glu81, and Trp174) in AmGSTO1, each was substituted with alanine via site-directed mutagenesis, and the kinetic parameters were measured. Replacement of Cys28 in the G-site (C28A) caused substantial changes in enzyme kinetics compared with the wild type. The *K*_m_ value for GSH was 0.03 mM in the wild-type AmGSTO1 but increased 9.67-fold in the C28A mutant, suggesting decreased binding affinity (Table 2). The *k*_cat_ value for the C28A mutant increased by 4.97-fold compared to that of the wild type. Its catalytic efficiency (*k*_cat_/*K*_m_) decreased by 0.51-fold compared to that of the wild type, suggesting impaired catalysis despite an increased turnover rate. Analysis of H-site mutants (Y30A, S82A, F127A, E81A, and W174A) revealed notable decreases in *k*_cat_ values. The greatest reduction in turnover was observed in the Y30A mutants (7.26-fold), followed by F127A (5.42-fold), S82A (4.17-fold), E81A (3.78-fold), and the W174A mutant (1.52-fold) (Table 2). All G-site and H-site mutants showed reduced catalytic efficiency (*k*_cat_/*k*_m_) relative to that of the wild-type enzyme, highlighting the importance of these residues for the catalytic activity of AmGSTO1.

### AmGSTO1 Binding to Selected Agrochemicals and Lack of Metabolic Transformation

Nonlinear regression analysis of ANS-AmGSTO1 saturation binding using a one-site binding model in GraphPad Prism 9.5.1 resulted in a *K*_d_ value of 50.41 ± 2.67 μM (Figure 6A). Various ligands and controls (GSH, CDNB, and EA) were evaluated as competitors of ANS bound at the H-site of AmGSTO1 (Table 3). As a negative control, GSH showed no inhibition, confirming its lack of interaction with the H-site (Figure 6B). In contrast, the positive controls, CDNB and EA, inhibited ANS binding, with IC_50_ values of 0.86 ± 0.02 and 1.70 ± 0.07 mM and had corresponding apparent *K*_i_ values of 434.34 and 858.59 μM, respectively (Figure 6B and Table 3). Among 38 ligands screened, six compounds inhibited ANS binding by competing for the AmGSTO1 H-site (Figure 7). 2,4-D, fenoprop, 3,5,6-trichloro-2-pyridinol (TCP), 3-phenoxybenzaldehyde, tetramethrin, and nicotine showed IC_50_ values of 7.43 ± 1.07, 5.73 ± 0.83, 4.43 ± 0.48, 3.80 ± 0.30, 0.13 ± 0.01, and 4.30 ± 0.41 mM, and corresponding apparent *K*_i_ values of 3752.53, 2893.94, 2237.37, 1919.19, 65.66, and 2171.72 μM, respectively (Figure 7 and Table 3). HPLC MS/MS was conducted to evaluate the potential metabolism of fenoprop, TCP, and 2,4-D by AmGSTO1. Incubations with active and heat-inactivated GST showed no significant differences (*p* > 0.05) (Figure 8), indicating that AmGSTO1 is unlikely to metabolize these compounds directly.

**Figure 6:**
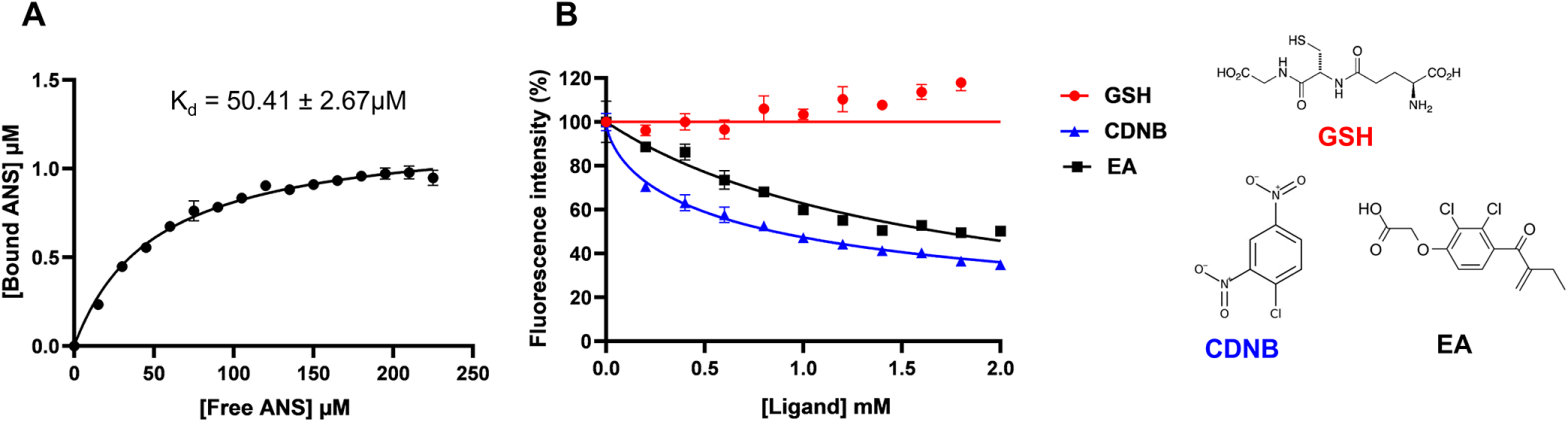
Fluorescence binding assay for AmGSTO1. (A) AmGSTO1 binding toward the fluorescent reporter 8-anilinonaphthalene-1-sulfonic acid (ANS). Samples containing 2 μM of AmGSTO1 were mixed with various concentrations of ANS in a buffer solution composed of 20 mM potassium phosphate, 150 mM NaCl, 1 mM EDTA, and 1 mM TCEP at pH 6. Fluorescence intensity was recorded using excitation and emission filters of 380 nm/20 nm and 485 nm/20 nm, respectively. Data analysis was performed using GraphPad Prism software with a one-site-specific binding model. (B) The displacement of ANS bound to AmGSTO1 was investigated using positive controls (CDNB and EA) and a negative control (GSH).

**Figure 7:**
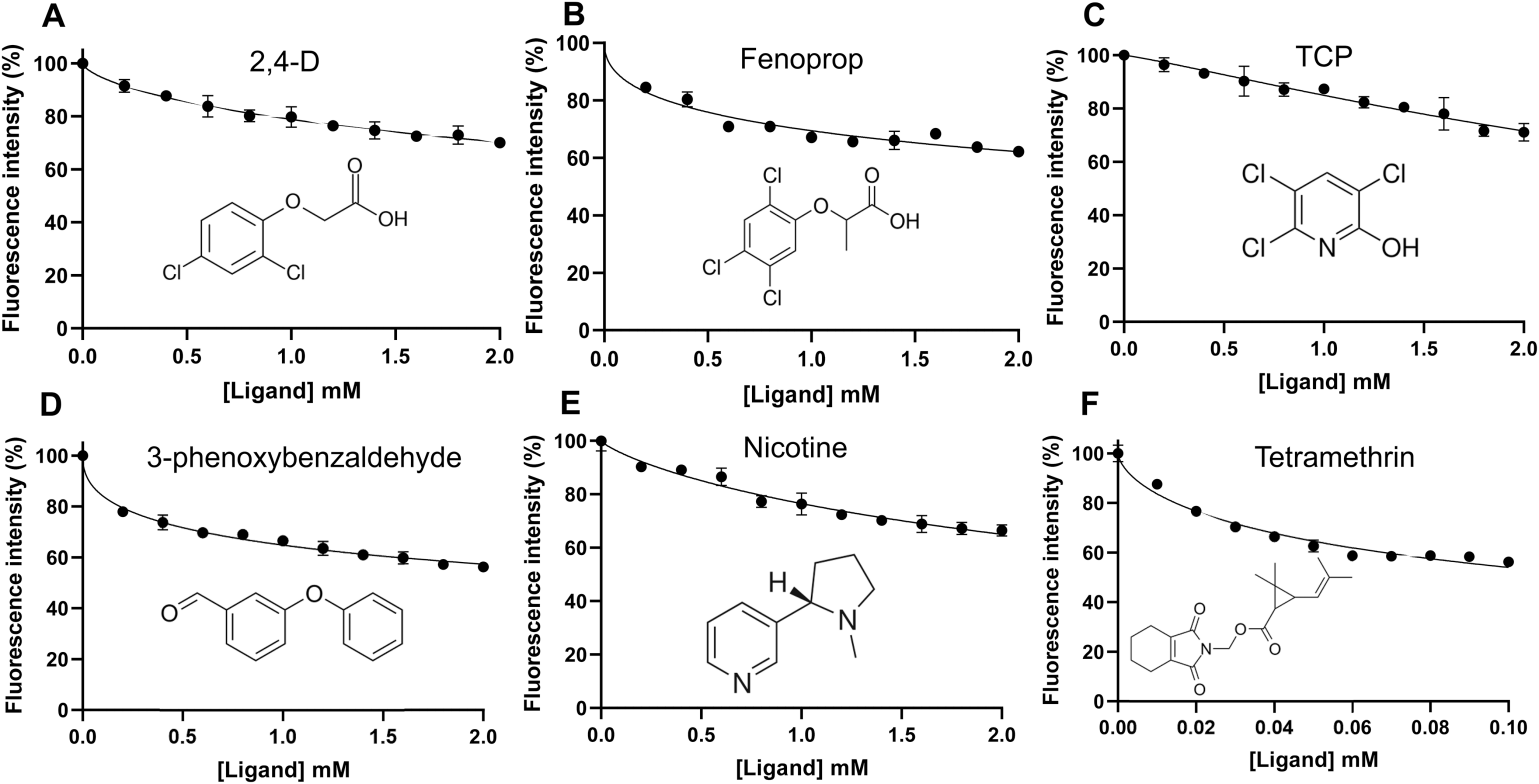
Displacement of ANS bound to AmGSTO1 was examined using competitive ligands, including (A) 2,4-D, (B) fenoprop, (C) TCP, (D) 3-phenoxybenzaldehyde, (E) nicotine, and (F) tetramethrin.

**Figure 8:**
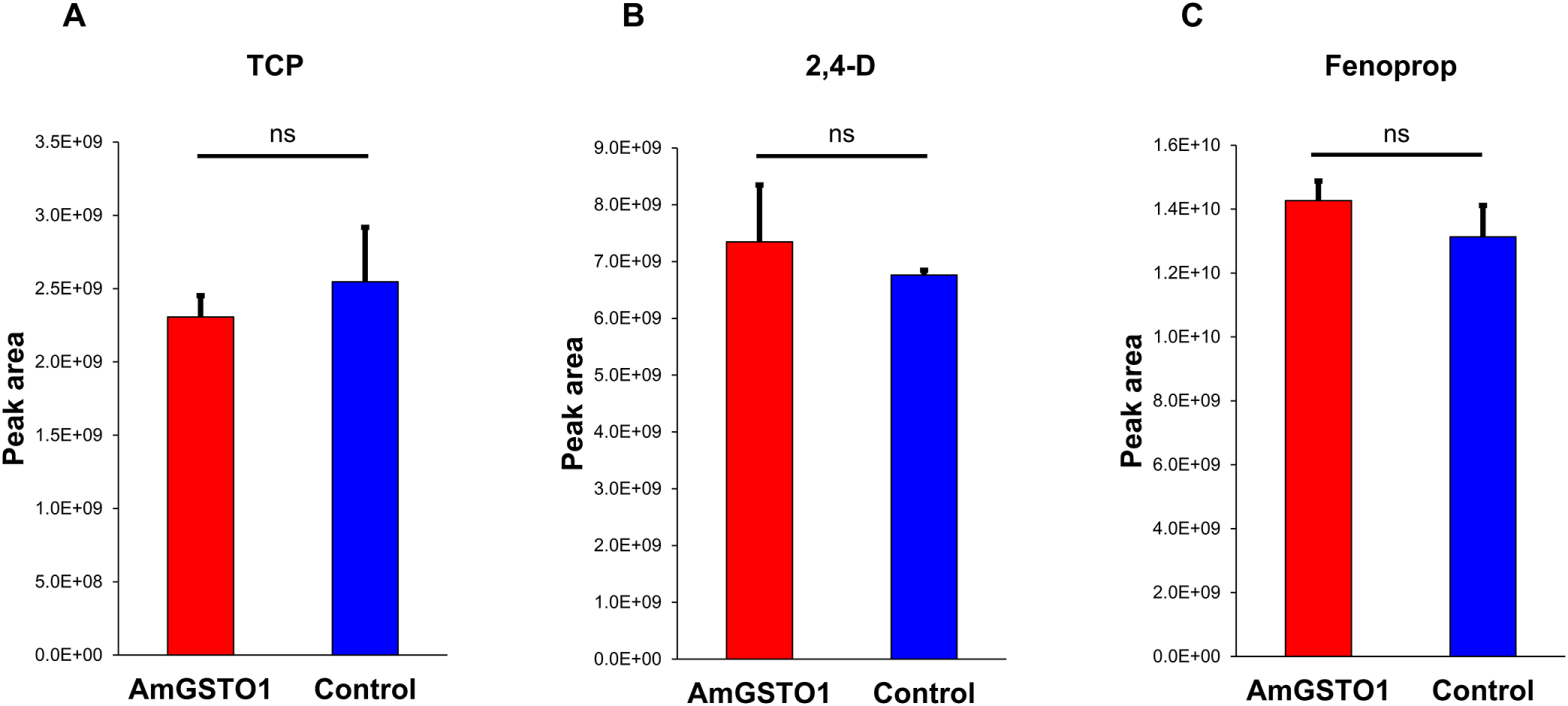
HPLC analysis of AmGSTO1 metabolic activity toward selected substrates. Bar graphs show chromatographic peak areas of (A) TCP, (B) 2,4-D, and (C) fenoprop detected in reaction mixtures containing active recombinant AmGSTO1 or heat-inactivated AmGSTO1 (control). Data are mean ± SD (n = 3). No significant differences were observed between treatments (*p* > 0.05).

**Table 3:** Competitive Binding Data of Different Ligands to AmGSTO1

category	ligand names	inhibition percentage (%)	IC_50_(mM)	*K*_i_(μM)
Controls	GSH	-	-	-
	CDNB	65.09	0.86 ± 0.02	434.34
	EA	49.72	1.70 ± 0.07	858.59
				
Herbicides	Atrazine	-	-	-
	Clopyralid	-	-	-
	2,4-D	29.96	7.43 ± 1.07	3752.53
	Dicamba	-	-	-
	Fenoprop	37.81	5.73 ± 0.83	2893.94
	Metolachlor	-	-	-
	Paraquat	-	-	-
	Picloram	-	-	-
	TCP	28.87	4.43 ± 0.48	2237.37
	Triclopyr	-	-	-
				
Fungicides	Chlorothalonil	-	-	-
	Myclobutanil	-	-	-
	Propiconazole	-	-	-
	Pyraclostrobin	-	-	-
				
Neonicotinoids	Dinotefuran	-	-	-
	Imidacloprid	-	-	-
	6-chloronicotinic acid	-	-	-
				
Carbamate	Carbaryl	-	-	-
				
Acaricides	Amitraz	-	-	-
				
Organophosphates	Acephate	-	-	-
	Coumaphos	-	-	-
	Chlorpyrifos	-	-	-
	Diazinon	-	-	-
	Malathion	-	-	-
	Metamidophos	-	-	-
				
Pyrethroids	Bifenthrin	-	-	-
	Deltamethrin	-	-	-
	Fenvalerate	-	-	-
	Permethrin	-	-	-
	3-Phenoxybenzaldehyde	43.74	3.80 ± 0.30	1919.19
	Tetramethrin	43.82	0.13 ± 0.01	65.66
				
Natural products	Caffeine	-	-	-
	Cotinine	-	-	-
	Nicotine	33.58	4.30 ± 0.41	2171.72
	p-Coumaric acid	-	-	-
	Phenethyl isothiocyanate	-	-	-
	Phenylacetaldehyde	-	-	-
	Propyl isothiocyanate	-	-	-

### Antioxidative Properties of AmGSTO1

Disc diffusion and bacterial survival assays were used to evaluate the antioxidative role of AmGSTO1 against oxidative stress. Cumene hydroperoxide (CHP), hydrogen peroxide (H_2_O_2_), and paraquat were used as oxidative stress inducers.^74^ Disc diffusion assays revealed significant differences between the pET-9Bc vector (control) and the recombinant AmGSTO1-pET-9Bc strains at all CHP (*p* < 0.001; df = 9; *F* = 396.54) and H_2_O_2_ concentrations (*p* < 0.001; df = 9; *F* = 295.15). After exposure to the CHP or H_2_O_2_, halo zone diameters were significantly smaller in LB agar plates containing AmGSTO1-expressing bacteria than in control plates (Figures 9A–C and S3). Compared with the control, exposure to the highest concentrations of CHP and H_2_O_2_ (200 mM) resulted in reductions of 43.33 and 41.18% in average halo diameters, respectively. To complement the disc diffusion assays, bacterial assays were performed to quantitatively assess the antioxidant function of AmGSTO1. Survival assays revealed that the cells expressing AmGSTO1 exhibited significantly higher tolerance to H_2_O_2_ and paraquat than control cells (Figure 10A,B). Increased OD_600 nm_ in the treatment group compared to the control indicated enhanced bacterial survival. Colony-forming unit (CFU) counts following H_2_O_2_ or paraquat exposure showed survival rates of 90.4 and 83.3%, respectively, in AmGSTO1-expressing cells, significantly higher than in controls (*p* < 0.001) (Figure 10C–F). Both disc diffusion and bacterial survival assays showed an increased oxidative tolerance in AmGSTO1-expressing bacterial cells.

**Figure 9:**
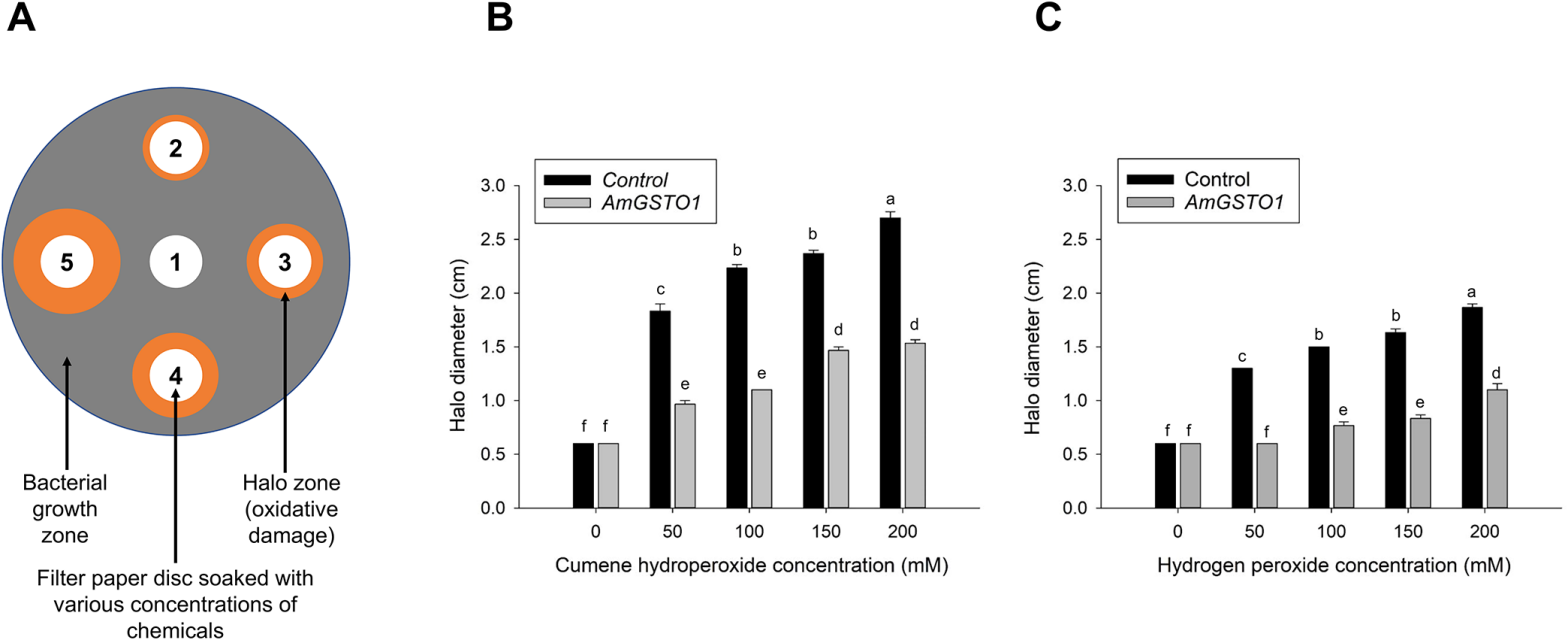
Disc diffusion assays in *E. coli* cells expressing AmGSTO1. (A) Cartoon image of the filter paper disc soaked with varied concentrations of an oxidative-inducing chemical, *E. coli* cell growth in an LB agar plate with antibiotics ampicillin (200 μg/mL) and chloramphenicol (30 μg/mL), and halo zone (clear area) created due to the killing of cells by chemicals. (B) The halo zone developed under various concentrations of cumene hydroperoxide. (C) The halo zone developed under various concentrations of hydrogen peroxide. Different letters indicate significant differences in halo zone formation between treatment and control as per one-way ANOVA with the Tukey HSD test.

**Figure 10:**
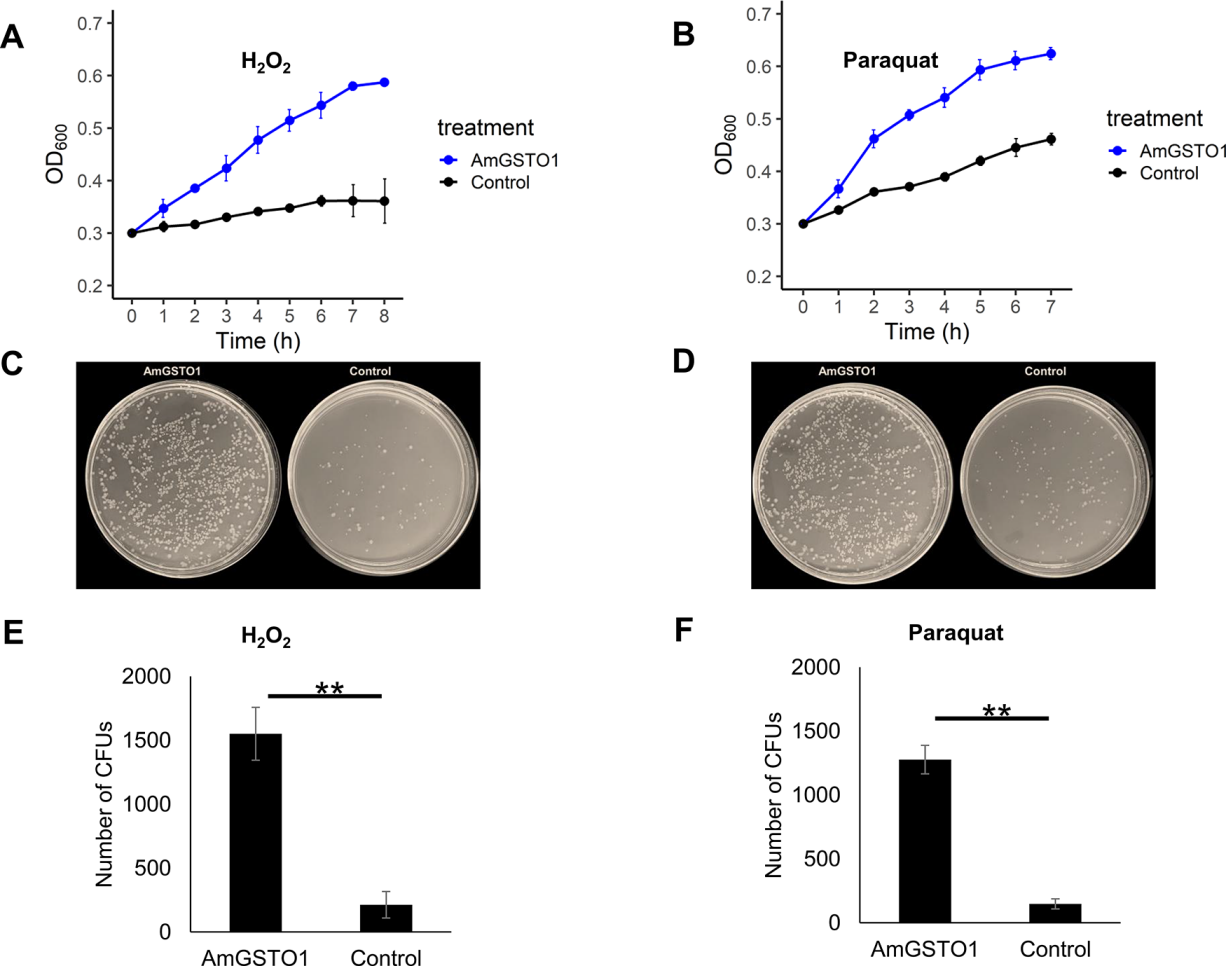
Bacterial survival assay in *E. coli* cells expressing AmGSTO1. *E. coli* cells consisting of recombinant AmGSTO1 and pET-9Bc vector were grown in 2YT media containing H_2_O_2_ and paraquat (0.5 mM). The OD_600_ of *E. coli* was measured every hour following exposure to H_2_O_2_ (A) and paraquat (B). After 7–8 h of incubation at 37 °C at 180 rpm, *E. coli* cells with recombinant AmGSTO1 and pET-9Bc vector were smeared on agar plates and grown at 37 °C overnight (C, D). The number of bacteria colony-forming units (CFUs) on the agar plates was recorded under the microscope (E, F). ** Indicates a significant difference between the two groups as per Student’s *t*-test analysis (*p* < 0.001).

## Discussion

GSTs are central to detoxification processes and are thought to contribute to chemical adaptation in a variety of arthropod species.^20,22,25,76–78^ In honey bees, GST genes have been identified in different tissues and linked to diverse functions, including xenobiotic detoxification and olfaction.^17,79–81^ Accumulating evidence from studies of omega-class GSTs in humans, microorganisms, and insects suggests that these enzymes contribute to oxidative stress mitigation.^73,82–85^ Our study characterized the structure and function of the omega-class GST, AmGSTO1, in honey bees. The findings presented here provide insights into potential mechanisms by which this enzyme could support honey bee adaptation to chemical stressors. In particular, AmGSTO1 may be involved in the sequestration of pesticides and plant allelochemicals and in mitigating oxidative stress, which will protect honey bees from diverse chemical stresses in their environment.

The cocrystal structure of AmGSTO1 with GSH revealed the amino acids forming the binding pocket. Our data showed that Cys28 in the G-site of AmGSTO1 is essential for GSH binding and activity with Cys28 forming a mixed disulfide bond with GSH in the cocrystal structure (Figure 4A,C). Moreover, the Cys28A mutation significantly reduced catalytic efficiency (Table 2). This decrease in catalytic efficiency was evident by an increased *K*_m_ value in the C28A mutant, indicating a reduced GSH affinity after mutation. Omega GSTs are characterized by a conserved cysteine residue within the G-site, which facilitates the formation of a disulfide bond with GSH, in contrast with other GST classes that typically employ tyrosine or serine residues at the active site, highlighting a distinct catalytic mechanism for omega GSTs.^26,73,82^ AmGSTO1 exhibited significant DHA reductase activity, with a DHA reductase specific activity (0.257 ± 0.002 μmol/min/mg) within the range reported for mammalian GSTO1 enzymes, falling between human GSTO1 (0.14 ± 0.013 μmol/min/mg) and rat GSTO1 (0.39 ± 0.08 μmol/min/mg), indicating a redox function as observed in other characterized omega GSTs.^60,64,86^ Additionally, AmGSTO1 displayed activity toward CDNB and PNA but showed substantially higher catalytic efficiency toward HED (0.36 ± 0.06 μM^–1^·min^–1^) compared with CDNB (7.76 ± 0.72 mM^–1^·min^–1^) and PNA (3.10 ± 0.66 mM^–1^·min^–1^) (Table 2). This preference for HED supports a thioltransferase function, consistent with reports for omega GSTs in insects, mammals, and fungi.^33,64,87^

In most omega GSTs, a proline residue commonly follows the conserved cysteine and is thought to stabilize the position of the cysteine thiol group, thereby supporting its chemical reactivity.^82,88^ Consistent with this pattern, Pro29 occurs immediately after Cys28 in AmGSTO1 (Figure 4C and S2). Additionally, Arg33 and Leu38 were identified as conserved residues within the G-site of AmGSTO1 (Figure 4C and S2), similar to those reported for omega GSTs in *Homo sapiens*.^89^ This conservation suggests that these amino acids may be critical for omega-GST functions across diverse species and may contribute to maintain structure integrity. Adjacent to the G-site, a set of residues form a hydrophobic pocket, referred to as the H-site (Figure 4B), which is thought to facilitate substrate binding and subsequent conjugation with GSH.^21,90^ Alanine substitution of the H-site residues Tyr30, Ser82, Phe127, Glu81, and Trp174 led to reduced *k*_cat_ and *k*_cat_/K_m_ values, highlighting their importance for functionally efficient catalysis. The decreases in turnover and catalytic efficiency of Tyr30, Phe127, and Trp174 to alanine mutations point to the role of aromatic residues in defining the hydrophobic and productive geometry of the H-site for electrophilic aromatic substrates (Table 2).

Previous studies have employed competitive binding assays with hydrophobic fluorescent probes (e.g., ANS) to screen the substrate spectrum and binding capabilities of GSTs.^28,44^ ANS binding studies showed that AmGSTO1 binds to the pesticides tetramethrin, 2,4-D, and fenoprop, to their metabolic byproducts: 3-phenoxybenzaldehyde and TCP, and to nicotine, a natural toxin found in *Nicotiana* pollen and nectar (Figure 7 and Table 3). The pesticide tetramethrin displayed the strongest binding out of the compounds tested, with an apparent *K*_i_ value of 64.21 μM. Pyrethroid insecticides are among the most frequently detected pesticide residues in beeswax, pollen, and honey bee-associated matrices in a previous study.^11^ Although pyrethroids are highly toxic to insects, including honey bees, their field application rates are typically low due to this potency and their reported repellent properties; however, increasing evidence indicates that even sublethal exposure can significantly impair bee physiology and behavior.^91,92^ The selective binding of AmGSTO1 towards tetramethrin, 2,4-D, fenoprop, 3-phenoxybenzaldehyde, TCP, and nicotine observed in this study (Figure 7 and Table 3) suggests a degree of substrate specificity that may reflect differences in molecular size, polarity, or metabolic transformation of the substrates. This pattern is consistent with the proposed role of omega-class GSTs in cellular defense processes, including antioxidant functions, rather than broad-spectrum xenobiotic conjugation.^85,93,94^ In disc diffusion and bacterial survival assays, heterologous expression of AmGSTO1 increased tolerance to oxidative stress induced by common reactive oxygen species (ROS) generators, including paraquat, CHP, and H_2_O_2_. Cells expressing AmGSTO1 exhibited improved growth and survival compared to control cells, suggesting that AmGSTO1 can enhance cellular resistance to oxidative stress in a bacterial model system (Figures 9 and 10). Although demonstrated in a heterologous system, these findings are consistent with a potential antioxidant function of AmGSTO1 in honey bees (*A. cerana cerana*) under chemical stress conditions.^93^

Besides insecticides, we also tested 10 commonly used herbicides and 4 fungicides (Table 3). Among them, two herbicides 2,4-D and fenoprop displayed 29.96 and 37.81% inhibition of ANS fluorescence, respectively, indicating their ability to interact with the hydrophobic binding site of AmGSTO1. TCP, a metabolite of herbicide triclopyr and a hydrolysis product of the organophosphate chlorpyrifos, also showed the ability to bind to AmGSTO1, exhibiting 28.87% inhibition of ANS fluorescence (Figure 7). Herbicides are generally considered to pose low acute toxicity to pollinators.^13^ However, exposure risk may increase substantially during the flowering period of crops or weeds attractive to bees. Elevated or repeated exposure has been reported to adversely affect bee health through mechanisms such as alterations in gut symbiont composition and disruption of colony-level processes, including collective thermoregulation.^95,96^ When combined, multiple pesticides, herbicides, and insecticides can produce synergistic or active effects that amplify toxicity and interfere with detoxification pathways, as reported in previous studies.^97–100^ In our previous study, we found that delta-class GST in honey bees (AmGSTD1) exhibits binding affinity with 2,4-D, triclopyr, and TCP, as well as some insecticides and fungicides.^44^ Together with the present findings for AmGSTO1, these results suggest that honey bee GSTs contribute to chemical tolerance by interacting with a broad range of agrochemicals. However, competitive binding alone does not necessarily indicate metabolic activity. Although classical GST activity involves glutathione conjugation, there is precedent for GSTs interacting with xenobiotics in ways that do not result in direct metabolism. For example, in the fruit pest *Cydia pomonella*, GSTs, including omega-class family members, were reported to bind the pyrethroid lambda-cyhalothrin without observable metabolic products, supporting the idea of sequestration in insecticide resistance.^101^ Similarly, an omega-class GST in *Anopheles cracens* was shown to bind the organophosphate insecticide temephos without detectable glutathione conjugation, indicating that these enzymes can interact with xenobiotics through mechanisms other than classical catalysis.^102^ Catalyzed reactions indicate metabolic activity, in which enzymatic transformation of the ligand yields products that are often less toxic and more water-soluble, thereby facilitating excretion.^103–105^ Our binding assay and *in vitro* enzymatic assay coupled with HPLC suggest that AmGSTO1 may be involved in the sequestration of fenoprop, 2,4-D, and TCP, rather than directly metabolizing these chemicals (Figure 8). Similar noncatalytic roles have been reported for omega-class GSTs in other species. For example, Ding et al. demonstrated that PcGSTO1 contributes to cyetpyrafen resistance through a noncatalytic passive binding and sequestration mechanism. HPLC analysis showed a dose-dependent reduction in cyetpyrafen residues, while the absence of detectable metabolites in MS analysis suggested that the enzyme functions as a molecular “sponge” rather than an active degradative catalyst.^37^ Likewise, Wongtrakul et al. reported that AcGSTO1-1 from *A. cracens* exhibited no catalytic metabolism of temephos, as evidenced by HPLC analysis, but displayed significant binding affinity for the insecticide. The absence of detectable metabolites, together with their binding capacity, supports a potential noncatalytic detoxification role. Collectively, these findings suggest that omega-class GSTs may contribute to insecticide resistance through sequestration mechanisms, representing a passive mode of detoxification as proposed in previous studies.^23,101,102,106^ From an evolutionary perspective, such a sequestration-based mechanism may represent an advantageous strategy for coping with chronic, low-dose chemical exposure. Unlike catalytic detoxification, which requires precise substrate recognition and can generate reactive intermediates, ligand sequestration provides a passive and energetically efficient means of reducing the bioavailability of xenobiotics. For generalist pollinators such as honey bee, which encounter a chemically diverse landscape of plant allelochemicals and anthropogenic agrochemicals, selective pressure may favor detoxification enzymes with broad binding capacity rather than narrow substrate specificity. The ability of AmGSTO1 to bind multiple herbicides and pesticide metabolites without catalyzing their transformation is consistent with this model and aligns with the known functional characteristics of omega-class GSTs, which are frequently associated with redox homeostasis and stress tolerance rather than extensive xenobiotic conjugation.^107^ Together, these features suggest that sequestration-based interactions mediated by GSTs may have been evolutionarily favored as a flexible and protective response to environmental chemical stress.

Interestingly, nicotine, an alkaloid naturally occurring in *Nicotiana* nectar and pollen as well as in botanical insecticides, showed binding affinity toward AmGSTO1, exhibiting 33.58% inhibition of ANS fluorescence (Figure 7). This interaction may help to explain, at least in part, the tolerance to nicotine-containing host plants.^108,109^ As an agonist of nicotinic acetylcholine receptors (nAChRs), nicotine has been proposed to act as a reward component in nectar, potentially influencing foraging behavior through its involvement in neural signaling.^109^ Previous studies have reported upregulation of GSTs in honey bees following nicotine exposure, suggesting potential roles in Phase I and/or II detoxification processes.^79^ Taken together, the observed binding of both synthetic pesticides and plant allelochemicals in this study suggests that AmGSTO1 possesses a degree of structural flexibility that enables selective interactions with chemically diverse ligands.

In conclusion, this study characterized the tissue-specific expression patterns of *AmGSTO1* in nurse and forager honey bees and demonstrated its antioxidative capacity. Structural analyses identified key amino acid residues that are likely important for the catalytic and functional properties of AmGSTO1. Importantly, our findings suggest that AmGSTO1 may contribute to honey bee adaptation to agrochemicals and plant allelochemicals through a combination of selective ligand binding and mitigation of oxidative stress. Although AmGSTO1 did not directly metabolize several tested compounds, its binding capacity and antioxidant function support a role in cellular defense against chemical stress. Together, these results provide new insights into the structure–function relationships of omega-class GSTs in the honey bee and advance our understanding of how insects cope with diverse xenobiotic challenges. Future *in vivo* approaches, such as gene knockdown or knockout, followed by survival assays are required to functionally validate the role of *AmGTSO1* in agrochemical tolerance in honey bees.

## Supplementary Material



## Notes

SDS-PAGE analysis of multistep chromatographic purification of recombinant AmGSTO1 (Figure S1); multiple sequence alignment of omega-class GSTs, highlighting conserved residues in honey bee (*Apis mellifera*) (AmGSTO1), silkworm (*Bombyx mori*) (BmGSTO), and human (*Homo sapiens*) (Figure S2); representative images of disc diffusion assays (Figure S3); primers used for this study (Table S1); insect GST sequence data for phylogenetic analysis (Table S2) (PDF)
