# Supplementary material for: Structural and Functional Insights into a Honey Bee Omega-Class Glutathione S‑Transferase Mediating Chemical Sequestration and Antioxidative Stress
Source: J Agric Food Chem. 2026 May 28;74(28):21881–98. doi: 10.1021/acs.jafc.6c03265 (PMC13397897; doi:10.1021/acs.jafc.6c03265)
Supplement: Supplementary file 1 [file jf6c03265_si_001.pdf]

Supplementary Information

Structural and functional insights into a honey bee Omega class glutathione S-transferase mediating chemical sequestration and antioxidative stress

Sonu Koirala[a], Timothy W. Moural[a,*], Gaurab Bhattarai[b], Ngoc T Phan[c,d], Edwin G Rajotte[a], David J Biddinger[a,e], Fang Zhu[a,f,*]

[a] Department of Entomology, Pennsylvania State University, University Park, PA 16802, USA

[b] Institute of Plant Breeding, Genetics & Genomics, University of Georgia, Athens, GA 30602, USA

[c] Department of Entomology and Plant Pathology, University of Arkansas, AR 72701, USA

[d] Research Center for Tropical Bees and Beekeeping, Vietnam National University of Agriculture, Gia Lam, Hanoi 100000, Viet Nam

[e] Penn State Fruit Research and Extension Center, Biglerville, PA 17307, USA

[f] Huck Institutes of the Life Sciences, Pennsylvania State University, University Park, PA 16802, USA

*Corresponding authors (fuz59@psu.edu and twm78@psu.edu)

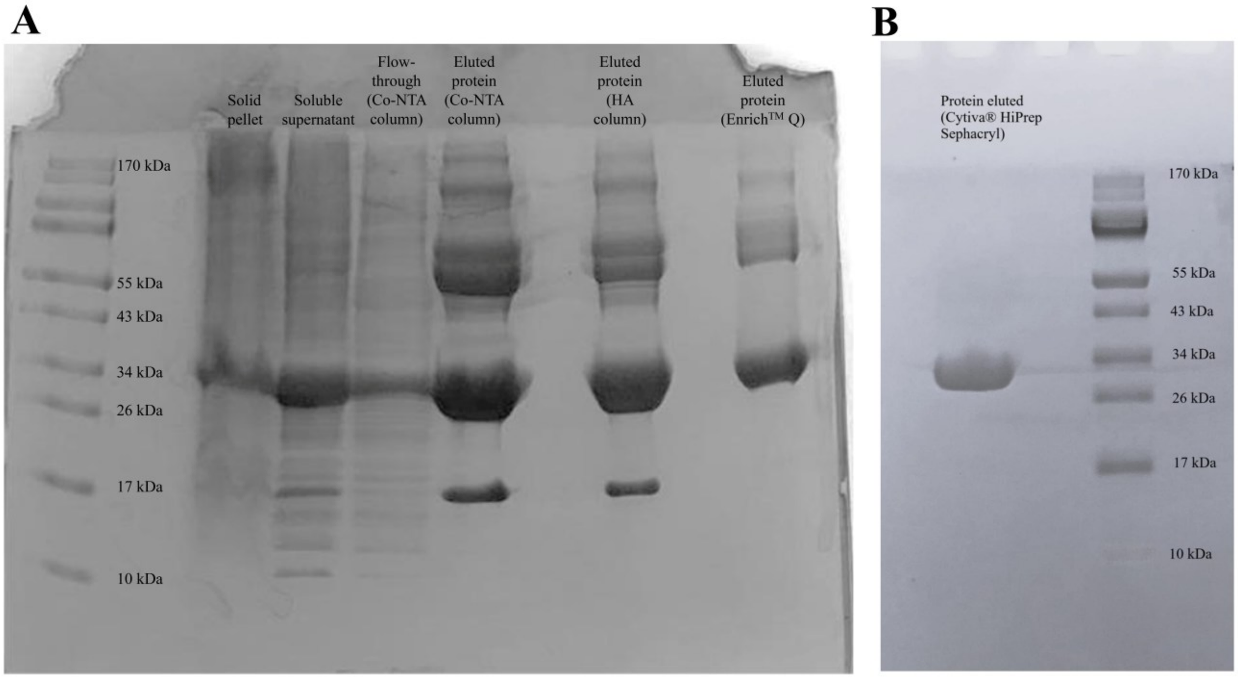

Fig. S1. SDS-PAGE analysis of recombinant AmGSTO1 purification. (A) SDS-PAGE analysis of AmGSTO1 purification. Lane order from left to right: molecular weight marker (kDa); proteins from solid pellet and soluble supernatant after lysis and centrifugation; Co-NTA column flow through and bound protein fractions; eluted protein obtained after HA column purification; and protein fraction from HA column purified using Enrich™ Q. (B) SDS-PAGE for final purified AmGSTO1. Lane order from left to right: protein fraction obtained from Enrich™ Q further purified using Cytiva® HisPrep Sephacryl column; and molecular weight marker (kDa). The molecular weight of AmGSTO1 is approximately 29.83 kDa.

```
AmGSTO1      -----------MSSKHLTIGSVAP-PIVPGKIRLYSMRFCPYAQRIHLVLDAKHIPHDVV 48
BmGSTO       MSAIKDSRNINFNTKHLRKG--DPLPPFNGKLRVYNMRYCPYAQRTILALNAKQIDYEVV 58
HsGSTO1      MS--------GESARSLGKGSAPPGPVPEGSIRIYSMRFCPFAERTRLVLKAKGIRHEVI 52
HsGSTO2      MS--------GDATRTLGKGSQPPGPVPEGLIRIYSMRFCPYSHRTRLVLKAKDIRHEVV 52
```

Fig. S2. Multiple sequence alignment of omega-class GSTs in honey bee (*Apis mellifera*) (AmGSTO1), silkworm (*Bombyx mori*) (BmGSTO), and human (*Homo sapiens sapiens*) (HsGSTO1 and HsGSTO2). Amino acids highlighted in green represent conserved amino acid residues at the G-site. HsGSTO1 (PDB: 1EEM_A), HsGSTO2 (PDB:3Q18_A), BmGSTO (PDB: 3WD6).

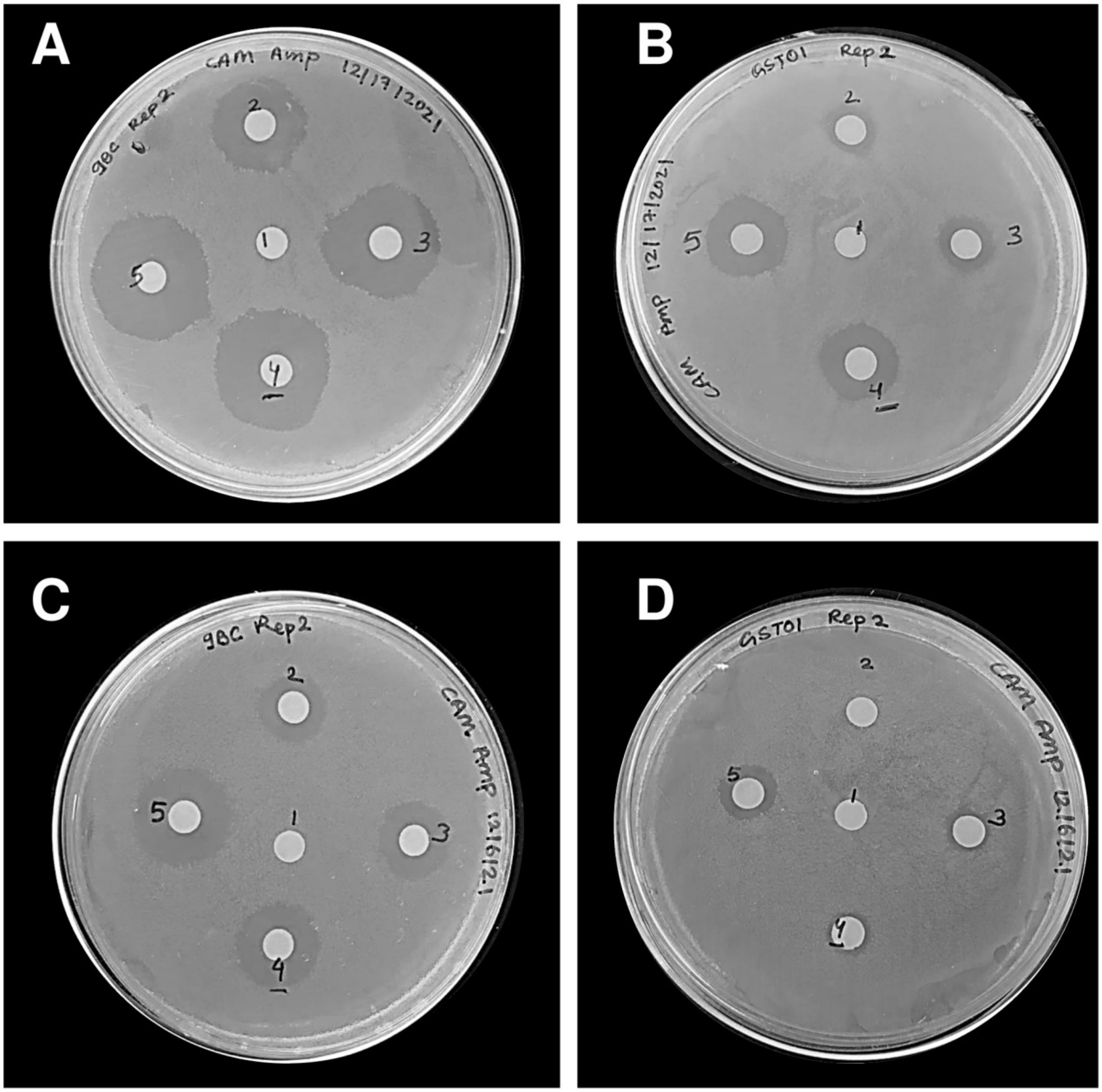

Fig. S3. Disc diffusion assay for AmGSTO1. The agar plates coated with bacterial cells expressing either pET-9BC or AmGSTO1 vector displayed qualitative observations of distinct halo zones when exposed to different concentrations of oxidative inducers. A and B: *E. coli* expressing pET-9BC and AmGSTO1, respectively, following exposure to cumene hydroperoxide; C and D: *E. coli* expressing pET-9BC and AmGSTO1, respectively, exposed to hydrogen peroxide.

Table S1. Primers used for this study, including qRT-PCR and full-length primers sequence for *AmGSTO1* as well as forward and reverse primers for creating mutations via substitution in active sites of GST genes. Nucleotide sequence with small letter indicates the codon targeted for mutation.

Sequence Name	Sequence (5'→ 3')	Purpose
qAmGSTO1F	ATTTGACTATCGGATCAGTTGC	qRT-PCR
qAmGSTO1R	CTTTGGGCATATGGACAAAATC	
qGAPDH-F	ACCTTCTGCAAAATTATGGCGA	
qGAPDH-R	ACCTTTGCCAAGTCTAACTGTTAAG	
FullAmGSTO1F	TTTAAGAAGGAGATATAGTTCATGAGTTCTAAACATTTGACTATC	Full length cloning and plasmid construction
FullAmGSTO1R	GGATTGGAAGTAGAGGTTCTCTTAATCAGTAATCAAATCATATTGT	
T7 promoter	TAATACGACTCACTATAGGG	
T7 terminator	GCTAGTTATTGCTCAGCGG	
K55A-F	TTTGACTCATgcgCCTGATTGGCTAC	Site-directed mutagenesis
K55A-F	TTTACATAAACAACATCATGAG	
C28A-F	TATGCGATTTgcgCCATATGCCCAAAG	
C28A-R	CTATATAAACGTATTTTTCCAG	
Y30A-F	ATTTTGTCCAgcgGCCCAAAGAATTCATTTAG	
Y30A-R	CGCATACTATATAAACGTATTTTTC	
S82A-F	ATTATATGAAgcgCTTGTAATTGCTGAATATTTG	
S82A-F	ATTTCTCCTCCTTCTAATTC	
F127A-F	GTGTAAGTTGgcgATTAATACATCTATAGATC	
F127A-R	ATAGTATTTATAACAGAATTAAATCTAC	
E81A-F	AATATTATATgcgAGTCTTGTAATTGCTG	
E81A-F	TCTCCTCCTTCTAATTCTATAC	
W174A-F	GATATGGCCAgcgTGGGAAAGATC	
W174A-R	ATAAAATCTAACATGCCAGG	

Table S2. Protein sequences for insect GSTs used to construct maximum likelihood (ML) phylogenetic tree.

**Accession number**	**Species**	**GST Class**
NP_001171499.1	*Apis melifera*	Delta
XP_006563394.1	*Apis melifera*	Delta
NP_001034042.1	*Drosophila melanogaster*	Delta
NP_524326.1	*Drosophila melanogaster*	Delta
NP_524912.1	*Drosophila melanogaster*	Delta
NP_788656.1	*Drosophila melanogaster*	Delta
NP_524913.1	*Drosophila melanogaster*	Delta
NP_524914.3	*Drosophila melanogaster*	Delta
NP_524915.1	*Drosophila melanogaster*	Delta
NP_525114.1	*Drosophila melanogaster*	Delta
NP_524916.1	*Drosophila melanogaster*	Delta
NP_650181.1	*Drosophila melanogaster*	Delta
NP_001287303.1	*Drosophila melanogaster*	Delta
NP_652713.1	*Drosophila melanogaster*	Delta
NP_001287302.1	*Drosophila melanogaster*	Delta
NP_650183.1	*Drosophila melanogaster*	Delta
NP_001138040.1	*Drosophila melanogaster*	Delta
XP_313050.3	*Anopheles gambiae*	Delta
XP_313048.1	*Anopheles gambiae*	Delta
XP_313049.1	*Anopheles gambiae*	Delta
XP_562680.2	*Anopheles gambiae*	Delta
XP_313052.1	*Anopheles gambiae*	Delta
XP_313667.3	*Anopheles gambiae*	Delta
XP_562132.1	*Anopheles gambiae*	Delta
XP_307500.3	*Anopheles gambiae*	Delta
XP_313059.1	*Anopheles gambiae*	Delta

XP_313664.3	*Anopheles gambiae*	Delta
XP_562676.3	*Anopheles gambiae*	Delta
XP_562690.2	*Anopheles gambiae*	Delta
XP_313058.1	*Anopheles gambiae*	Delta
XP_313668.1	*Anopheles gambiae*	Delta
XP_313665.4	*Anopheles gambiae*	Delta
XP_313666.2	*Anopheles gambiae*	Delta
XP_974273.1	*Tribolium castaneum*	Delta
XP_015834775.1	*Tribolium castaneum*	Delta
XP_974204.1	*Tribolium castaneum*	Delta
NP_001037183.1	*Bombyx mori*	Delta
XP_037867589.1	*Bombyx mori*	Delta
XP_037867588.1	*Bombyx mori*	Delta
XP_037867590.1	*Bombyx mori*	Delta
NP_001036974.1	*Bombyx mori*	Delta
NP_001037546.1	*Bombyx mori*	Delta
XP_012546385.1	*Bombyx mori*	Delta
XP_037877194.1	*Bombyx mori*	Delta
XP_037877195.1	*Bombyx mori*	Delta
XP_037877196.1	*Bombyx mori*	Delta
XP_037877197.1	*Bombyx mori*	Delta
XP_037877198.1	*Bombyx mori*	Delta
XP_037877199.1	*Bombyx mori*	Delta
XP_037877201.1	*Bombyx mori*	Delta
XP_037877202.1	*Bombyx mori*	Delta
NP_611323.1	*Drosophila melanogaster*	Epsilon
NP_611324.1	*Drosophila melanogaster*	Epsilon
NP_611325.2	*Drosophila melanogaster*	Epsilon
NP_611326.1	*Drosophila melanogaster*	Epsilon

NP_611327.1	*Drosophila melanogaster*	Epsilon
NP_611328.1	*Drosophila melanogaster*	Epsilon
NP_611329.1	*Drosophila melanogaster*	Epsilon
NP_001286571.1	*Drosophila melanogaster*	Epsilon
NP_611330.2	*Drosophila melanogaster*	Epsilon
NP_725784.1	*Drosophila melanogaster*	Epsilon
NP_001286570.1	*Drosophila melanogaster*	Epsilon
NP_611322.1	*Drosophila melanogaster*	Epsilon
NP_001286575.1	*Drosophila melanogaster*	Epsilon
NP_611339.1	*Drosophila melanogaster*	Epsilon
NP_611964.1	*Drosophila melanogaster*	Epsilon
NP_001246500.1	*Drosophila melanogaster*	Epsilon
NP_001246501.1	*Drosophila melanogaster*	Epsilon
NP_001286857.1	*Drosophila melanogaster*	Epsilon
NP_001188889.1	*Drosophila melanogaster*	Epsilon
NP_610457.1	*Drosophila melanogaster*	Epsilon
NP_610855.1	*Drosophila melanogaster*	Epsilon
XP_319969.1	*Anopheles gambiae*	Epsilon
XP_319968.3	*Anopheles gambiae*	Epsilon
XP_319972.1	*Anopheles gambiae*	Epsilon
XP_319967.1	*Anopheles gambiae*	Epsilon
XP_319966.1	*Anopheles gambiae*	Epsilon
XP_001238234.1	*Anopheles gambiae*	Epsilon
XP_319970.3	*Anopheles gambiae*	Epsilon
XP_319963.1	*Anopheles gambiae*	Epsilon
XP_008200937.1	*Tribolium castaneum*	Epsilon
XP_015838206.1	*Tribolium castaneum*	Epsilon
XP_015838196.1	*Tribolium castaneum*	Epsilon
XP_015838248.1	*Tribolium castaneum*	Epsilon

XP_966787.1	*Tribolium castaneum*	Epsilon
XP_015838193.1	*Tribolium castaneum*	Epsilon
XP_966872.2	*Tribolium castaneum*	Epsilon
XP_966966.2	*Tribolium castaneum*	Epsilon
XP_008200938.1	*Tribolium castaneum*	Epsilon
XP_967147.1	*Tribolium castaneum*	Epsilon
XP_015838218.1	*Tribolium castaneum*	Epsilon
XP_967234.1	*Tribolium castaneum*	Epsilon
XP_967313.1	*Tribolium castaneum*	Epsilon
XP_967395.1	*Tribolium castaneum*	Epsilon
XP_971136.1	*Tribolium castaneum*	Epsilon
XP_971203.1	*Tribolium castaneum*	Epsilon
XP_008190708.1	*Tribolium castaneum*	Epsilon
XP_015833061.1	*Tribolium castaneum*	Epsilon
XP_971389.1	*Tribolium castaneum*	Epsilon
XP_015833061.1	*Tribolium castaneum*	Epsilon
XP_971509.1	*Tribolium castaneum*	Epsilon
NP_001037197.1	*Bombyx mori*	Epsilon
NP_001037420.1	*Bombyx mori*	Epsilon
NP_001108466.1	*Bombyx mori*	Epsilon
NP_001108460.1	*Bombyx mori*	Epsilon
NP_001108464.1	*Bombyx mori*	Epsilon
NP_001108465.1	*Bombyx mori*	Epsilon
XP_012548743.1	*Bombyx mori*	Epsilon
XP_037873490.1	*Bombyx mori*	Epsilon
XP_037873489.1	*Bombyx mori*	Epsilon
XP_037873488.1	*Bombyx mori*	Epsilon
NP_001153742.1	*Apis melifera*	Sigma
XP_026295805.1	*Apis melifera*	Sigma

NP_001136128.1	*Apis melifera*	Sigma
XP_006560566.1	*Apis melifera*	Sigma
NP_001261040.1	*Drosophila melanogaster*	Sigma
NP_725653.1	*Drosophila melanogaster*	Sigma
NP_523767.2	*Drosophila melanogaster*	Sigma
NP_725654.1	*Drosophila melanogaster*	Sigma
XP_311546.4	*Anopheles gambiae*	Sigma
XP_015832951.1	*Tribolium castaneum*	Sigma
XP_970714.1	*Tribolium castaneum*	Sigma
XP_008190960.1	*Tribolium castaneum*	Sigma
XP_967475.1	*Tribolium castaneum*	Sigma
XP_015833288.1	*Tribolium castaneum*	Sigma
XP_008191064.1	*Tribolium castaneum*	Sigma
XP_008191063.1	*Tribolium castaneum*	Sigma
XP_969146.1	*Tribolium castaneum*	Sigma
XP_970442.1	*Tribolium castaneum*	Sigma
NP_001037077.1	*Bombyx mori*	Sigma
NP_001036994.1	*Bombyx mori*	Sigma
XP_006569695.1	*Apis melifera*	Omega
NP_648237.1	*Drosophila melanogaster*	Omega
NP_729388.1	*Drosophila melanogaster*	Omega
NP_648236.1	*Drosophila melanogaster*	Omega
NP_648234.1	*Drosophila melanogaster*	Omega
XP_315763.2	*Anopheles gambiae*	Omega
XP_971118.1	*Tribolium castaneum*	Omega
XP_971184.1	*Tribolium castaneum*	Omega
XP_971247.1	*Tribolium castaneum*	Omega
NP_001040131.1	*Bombyx mori*	Omega
NP_001037406.1	*Bombyx mori*	Omega

NP_001040435.1	*Bombyx mori*	Omega
NP_001108461.1	*Bombyx mori*	Omega
XP_624692.2	*Apis melifera*	Theta
NP_610509.2	*Drosophila melanogaster*	Theta
NP_724816.3	*Drosophila melanogaster*	Theta
NP_001285495.1	*Drosophila melanogaster*	Theta
NP_001162808.1	*Drosophila melanogaster*	Theta
NP_728347.1	*Drosophila melanogaster*	Theta
NP_572886.2	*Drosophila melanogaster*	Theta
XP_311299.1	*Anopheles gambiae*	Theta
XP_003437127.1	*Anopheles gambiae*	Theta
XP_008196475.1	*Tribolium castaneum*	Theta
XP_015837903.1	*Tribolium castaneum*	Theta
NP_001108463.1	*Bombyx mori*	Theta
XP_012546617.1	*Bombyx mori*	Theta
XP_021204265.2	*Bombyx mori*	Theta
XP_016767705.1	*Apis melifera*	Zeta
NP_649894.1	*Drosophila melanogaster*	Zeta
NP_731358.1	*Drosophila melanogaster*	Zeta
NP_996190.1	*Drosophila melanogaster*	Zeta
NP_649895.1	*Drosophila melanogaster*	Zeta
XP_003436289.1	*Anopheles gambiae*	Zeta
XP_008195660.1	*Tribolium castaneum*	Zeta
XP_973541.3	*Tribolium castaneum*	Zeta
NP_001037418.1	*Bombyx mori*	Zeta
NP_001040453.1	*Bombyx mori*	Zeta
XP_309135.1	*Anopheles gambiae*	Unclassified
XP_307765.1	*Anopheles gambiae*	Unclassified
XP_310012.1	*Anopheles gambiae*	Unclassified

XP_975048.1	*Tribolium castaneum*	Unclassified
XP_970577.1	*Tribolium castaneum*	Unclassified
NP_001108462.1	*Bombyx mori*	Unclassified
NP_001040130.1	*Bombyx mori*	Unclassified
XP_394313.1	*Apis melifera*	Microsomal
XP_001119967.1	*Apis melifera*	Microsomal
NP_001285507.1	*Drosophila melanogaster*	Microsomal
NP_728382.1	*Drosophila melanogaster*	Microsomal
NP_524696.2	*Drosophila melanogaster*	Microsomal
NP_788903.1	*Drosophila melanogaster*	Microsomal
NP_001285264.1	*Drosophila melanogaster*	Microsomal
NP_001285263.1	*Drosophila melanogaster*	Microsomal
NP_788904.1	*Drosophila melanogaster*	Microsomal
XP_968617.1	*Tribolium castaneum*	Microsomal
XP_015836904.1	*Tribolium castaneum*	Microsomal
XP_015836906.1	*Tribolium castaneum*	Microsomal
XP_969139.2	*Tribolium castaneum*	Microsomal
